# Functional heterogeneity of cancer-associated fibroblasts with distinct neoadjuvant immunotherapy plus chemotherapy response in esophageal squamous cell carcinoma

**DOI:** 10.1186/s40364-024-00656-z

**Published:** 2024-09-27

**Authors:** Jun Jiang, Chao Xu, Donghui Han, Yuan Lu, Fa Yang, Jiawei Wang, Xiaolong Yan, Xiaorong Mu, Jipeng Zhang, Chenghui Jia, Xinyao Xu, Kui Liu, Zhenhua Liu, Li Gong, Yi Wan, Qiang Lu

**Affiliations:** 1 Department of Health Service, Base of Health Service, Air Force Medical University, Xi’an, China; 2 Department of Urology, Xijing Hospital, Air Force Medical University, Xi’an, China; 3 Department of Respiratory and Critical Care Medicine, Zhongda Hospital, Southeast University, Nanjing, China; 4 Department of Clinical Immunology, PLA Specialized Research Institute of Rheumatology & Immunology, Xijing Hospital, and National Translational Science Center for Molecular Medicine, Air Force Medical University, Xi’an, China; 5 Department of Thoracic Surgery, Tangdu Hospital, Air Force Medical University, NO. 569 Xinsi Road, Xi’an, 710038 China; 6 Department of Pathology, Department of Pharmacy, Tangdu Hospital, Air Force Medical University, NO. 569 Xinsi Road, Xi’an, 710038 China; 7 Department of Thoracic Surgery, The First Affiliated Hospital, Xi’an Medical College, Xian, China; 8 College of Life Sciences, Northwest University, Xian, China

**Keywords:** Esophageal squamous-cell carcinoma, Tumor microenvironment, Cancer-associated fibroblasts, Neoadjuvant immunochemotherapy, scRNA-seq

## Abstract

Novel neoadjuvant immunotherapy combined with chemotherapy (neoICT) has improved outcomes for patients with esophageal squamous-cell carcinoma (ESCC), but challenges persist in low response rates and therapy resistance. Little is known about the intra-tumoral heterogeneity in the ESCC tumor microenvironment (TME) that underlies differential responses to neoadjuvant therapy. We applied single-cell RNA sequencing (scRNA-seq) profiling and multiplexed immunofluorescence staining to thoroughly decipher the TME in ESCC specimens from a neoadjuvant anti-PD1 combination therapy clinical trial. The cancer-associated fibroblasts (CAFs) population showed the significant alteration in abundance following neoadjuvant therapy. Specifically, IL6 + CCL2 + immunomodulatory CAFs and a novel CD248 + mechanoresponsive CAFs subset exhibited increasing infiltration. Mechanistically, CD248 + mechanoresponsive CAFs approached and lined the tumor nest to physically block the infiltration of CD8 + T cells and drug delivery, while IL6 + CCL2 + immunomodulatory CAFs induced therapeutic resistance with distinct IL-6 expression. Among patients treated with neoICT, we observed prominent CAF-T cell interactions. In particular, the NECTIN2-TIGIT ligand-receptor pair was enriched in treated samples, and TIGIT was identified as the major inhibitory checkpoint of T cells. Our findings demonstrate distinct alterations in TME constituent responses to neoadjuvant immunotherapy and identify functional phenotypes of CAFs associated with unfavorable therapeutic responses in patients. This provides potential targets to enhance responses to neoadjuvant therapy in ESCC.

**Supplementary Information:**

The online version contains supplementary material available at 10.1186/s40364-024-00656-z.

## Introduction

The incidence rate of esophageal cancer (EC) is growing rapidly, and EC remains the sixth leading cause of cancer death worldwide [1]. ESCC accounts for approximately 85% of EC cases, with a 5-year survival rate for patients varying from 15 to 25% [2, 3]. Immunotherapy, either alone or combined with chemotherapy, has shown promising antitumor activity and safety in patients with advanced ESCC. It has also begun to be explored in the perioperative treatment of early-stage ESCC patients [4, 5]. A previous clinical trial, a prospective, single-arm, phase II study (TD-NICE), has elucidated that tislelizumab combined with carboplatin and nab-paclitaxel as neoICT exhibits clinically promising antitumor activity for resectable ESCC, with high rates of pathological complete response (pCR) and R0 resection [6]. However, the intratumor heterogeneity and complexity of TME contributing to differential clinical responses remain elusive. Therefore, exploring the underlying mechanisms and functions of tumor-infiltrating cells in the TME of ESCC is essential.

CAFs are predominant components of the TME [7]. Accumulating evidence indicates that CAFs are a heterogeneous and plastic population with distinct functions, including extracellular matrix (ECM) remodeling, metabolic reprogramming, immune cell exclusion, and drug resistance manipulation. They serve either pro-tumoral or anti-tumoral roles in cancer occurrence and progression [8–10]. CAFs are becoming important targets for cancer treatment. The origins and characterization of CAFs are complex and inconclusive [11]. With the application of scRNA-seq, distinct CAF populations and functions have gradually been unveiled in various cancers, including breast [12], pancreatic [13], lung [14, 15], head and neck [16], and colorectal cancer [17]. CAFs are often divided by their functional characteristics into three distinct subtypes: myofibroblastic/extracellular matrix CAFs (myCAFs), inflammatory CAFs (iCAFs), and antigen-presenting CAFs (apCAFs). Recently, a widely embraced framework for naming fibroblast subtypes has yet to emerge due to the diversity across multiple types of cancer [18]. Foster and colleagues have established a nomenclature system that defines three broad groups of CAFs across species and tissue types: steady-state-like (SSL), mechanoresponsive (MR), and immunomodulatory (IM) CAFs [19].

MR-CAFs have high mechanosensitive signaling mediators and ECM components, such as ACTA2, POSTN, and COL1A1. The ECM, composed of collagen, proteoglycans, and glycoproteins, is a highly dynamic architecture within the tumor stroma. CAFs play vital roles in mediating ECM remodeling by producing specific enzymes such as matrix metalloproteinase-1 and -3 (MMP-1 and MMP-3) [20]. Additionally, TGF-β is one of the most important mediators in ECM remodeling [21]. Dysregulations of the ECM by CAFs account for architectural and stiffness alterations contributing to tumor progression. IM-CAFs, a subset of CAFs opposite to MR-CAFs, express low levels of ECM but high levels of inflammation-related mediators, such as IL-6, ILR1, and CXCL12 [15]. SSL-CAFs, identified by Buechler et al. across multiple tissue types, express conservative genes such as PI16 and DPT, maintaining their quiescent fibroblast states [22].

However, the diversity of CAF subsets in ESCC and their discrete functional contributions to the TME and response to neoadjuvant therapy have not been disclosed. Here, we investigated the dynamic atlas of the ESCC TME landscape by performing scRNA-seq on 41 samples from 18 individuals in a novel neoadjuvant trial, TD-NICE, involving tislelizumab combined with carboplatin and nab-paclitaxel. We characterized the stromal cell compartment in ESCC through unsupervised clustering and identified six distinct CAF populations integrated into three superclusters: SSL-, MR-, and IM-CAFs. We revealed the radically different responses of CAF subsets to neoadjuvant therapy. This work also disclosed the interactions between the infiltration of CAF subsets and immunosuppressive T cells, helping to characterize the cellular and molecular dynamics driven by neoadjuvant therapy. These results could help predict clinical responsiveness or resistance to neoadjuvant therapy in ESCC patients and serve as potential therapeutic targets for future clinical trials.

## Results

### Overview of ESCC ecosystem analyzed by scRNA-seq

**Fig. 1:**
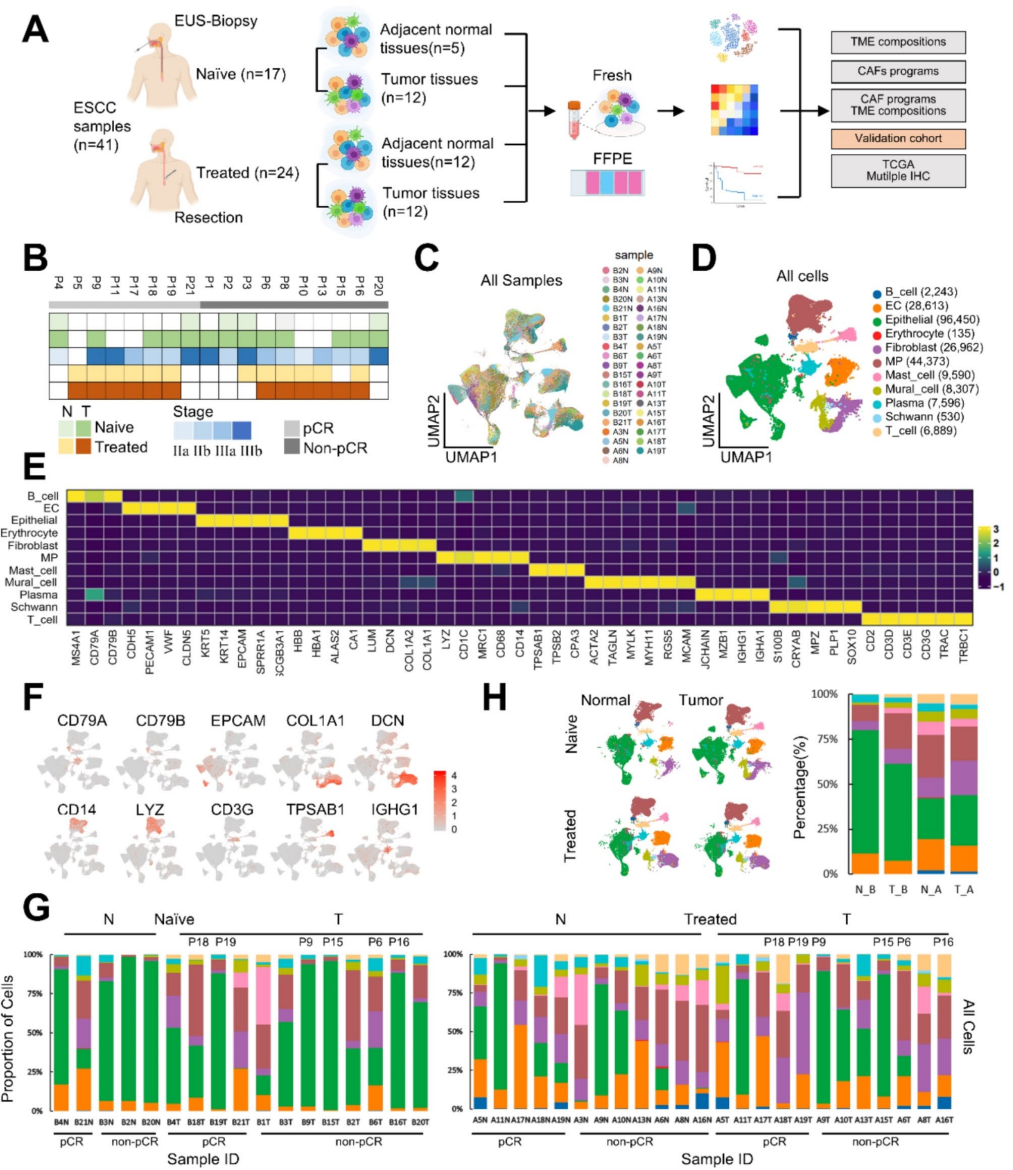
Overview of ESCC ecosystem characterized by scRNA-seq. (**A**) Workflow depicting sample acquisition, processing, and analysis. TME, tumor microenvironment; CAF, cancer-associated fibroblast; FFPE, formalin-fixed paraffin-embedded. (**B**) Clinicopathologic characteristics for each patient sample including treatment status, stage, and procedure. EUS, endoscopic ultrasound. (**C**) UMAP embedding of the expression profile of 41 samples, including 17 treatment-naïve endoscopic biopsies and 24 post-neoadjuvant combination therapy surgical resected specimens. Distinct clusters are annotated and color-coded. UMAP uniform manifold approximation and projection. (**D**) UMAP embedding of single-nucleus profiles of 232,710 cells. EC endothelial cell, MP, mononuclear phagocyte. Color coded by major cell type. (**E**) Heatmap of the five most differentially expressed genes within each cluster. (**F**) Expression levels of selected known marker genes across 232,710 unsorted cells illustrated in UMAP plots from both normal and tumor tissue in treatment-naïve and treated ESCC patients. (**G**) 11 cell types distributions stratified by treatment status, tissue type and pathological response across 41 samples. Proportions (y axis) of cell subsets (color legend, shared with panel A) across naive (*n* = 17) (left) versus treated (*n* = 24) (right). (**H**) UMAP embeddings (left) and the proportion of each cluster (right) stratified by treatment status and tissue type. N_B, normal tissues in treatment-naïve samples, T_B, tumor tissues in naïve samples, N_A, normal tissues in treated samples, T_A, tumor tissues in treated samples. Color legend, shared with panel A. Source data are provided as a Source Data file

To explore the cell composition in the ESCC TME before and after neoICT, we conducted a longitudinal scRNA-seq analysis of 41 specimens from 18 individuals. Tumor and adjacent normal tissues were obtained from pre-therapeutic biopsies and post-therapeutic surgical samples. We then immediately performed scRNA-seq using the 10x Genomics platform (Fig. 1A). After quality control for sequencing depth and mitochondrial gene read count, standardized, filtered data were used for cell clustering and annotation via principal component analysis (PCA). The clinicopathologic characteristics of each individual, including treatment status, stage, and procedure, are listed (Fig. 1B, table S1).

We obtained a total of 232,710 cell transcriptomes, including 114,512 cells from treatment-naïve samples (84,297 from tumor tissues and 30,125 from adjacent normal tissues) and 118,198 cells from treated samples (60,095 from tumor tissues and 58,103 from adjacent normal tissues). We identified 11 distinct cell types based on known gene signatures (Fig. 1C-F), including epithelial cells, mononuclear phagocytes, fibroblasts, endothelial cells, B cells, T cells, plasma cells, mast cells, schwann cells, mural cells and erythrocytes.

Although 11 distinct cell types were present in tissues derived from both treatment-naïve and treated patients, the infiltration of specific cell types in each sample varied. This variation may elucidate the heterogeneous cell infiltrations for diverse therapy responses (Fig. 1G and Fig. S1A). We confirmed that the malignant cells were copy number-unstable, while other stromal cells were copy number-stable via inferCNV analysis (Fig.S1B). To further assess the clinical relevance of the infiltration of diverse cellular populations to the neoICT response, we stratified UMAP profiles and cellular subtypes not only by treatment status but also in comparison with tumor or adjacent normal tissue (Fig. 1H, left panel). As expected, multiple cellular populations were altered after neoICT. There was a notable reduction in the proportion of epithelial cells following therapy, while stromal compositions, including fibroblast, immune cell, and endothelial cell populations, increased (Fig. 1H, right panel; Fig.S1C, Mann-Whitney U test).

### TME components are differentially modulated by neoadjuvant therapy

**Fig. 2:**
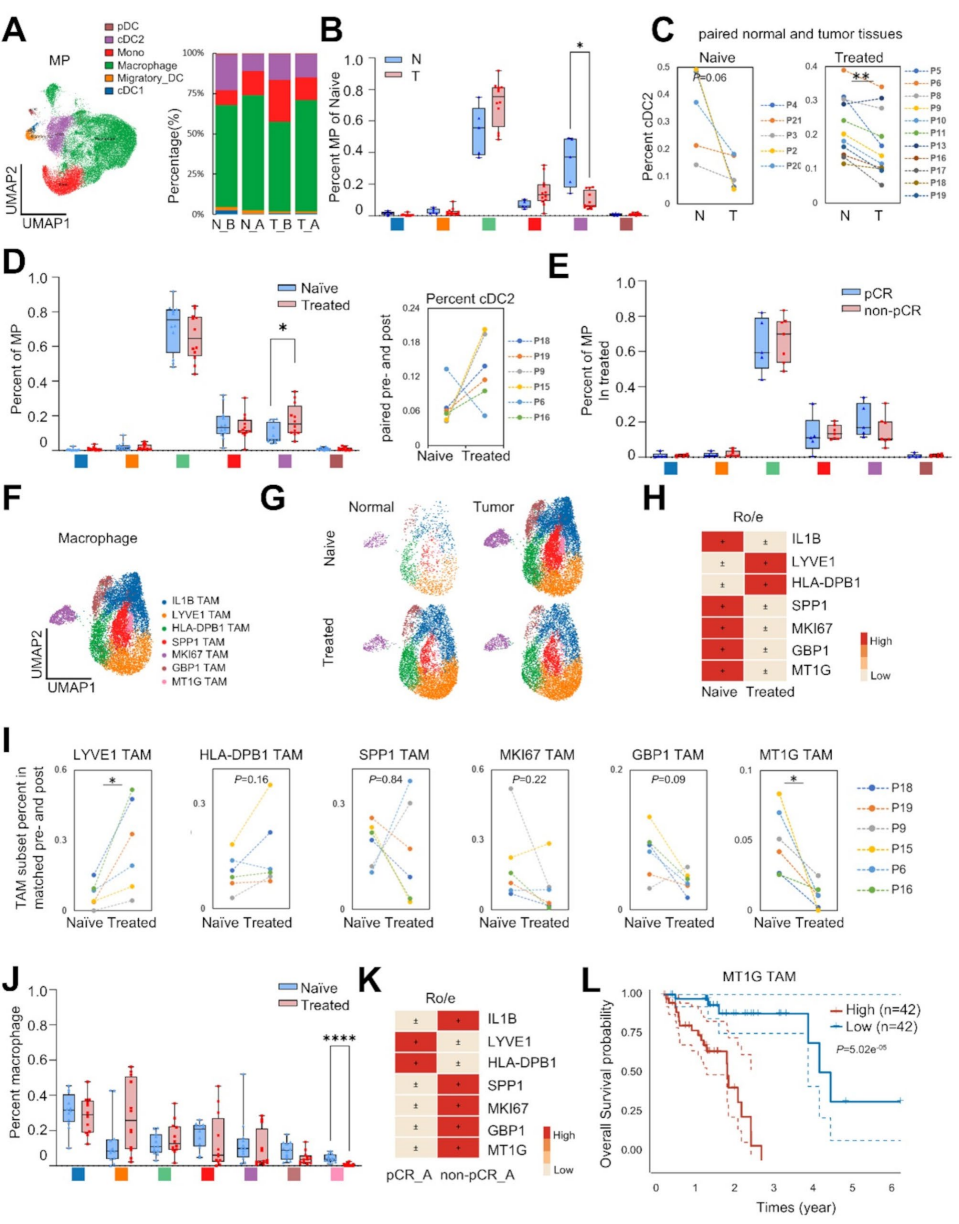
The cellular heterogeneity of mononuclear phagocytes compartment within the tumor microenvironment of ESCC pre- and post-neoICT. (**A**) UMAP plot of sub-clusters of mononuclear phagocytes (left). The proportion of each cluster split by treatment status and tissue type (right). pDC, plasmacytoid DC; cDC, conventional DC; Mono, monocytic cell. (**B**) Comparison of mononuclear phagocytes clusters of adjacent normal (N) vs. Tumor (T) in naïve samples by Mann-Whitney U test, * *P* < 0.05. (**C**) Comparison of cDC2% stratified by tissue type and treatment status by Wilcoxon matched-paired signed rank test, ** *P* < 0.01. 5 paired naïve patients, 12 paired treated patients with adjacent normal and tumor tissue. (**D**) Comparison of mononuclear phagocytes cluster proportions in naïve and treated samples (left), *P* values determined by Mann-Whitney U test. Comparison of cDC2% in 6 paired naïve and treated patients (right) by Wilcoxon matched-paired signed rank test, * *P* < 0.05. (**E**) Comparison of mononuclear phagocytes cluster proportions in differential pathological response by Mann-Whitney U test. pCR complete pathological response. (**F**) UMAP plot of 7 clusters of macrophages. (**G**) UMAP embedding of 7 macrophage clusters stratified by treatment and tissue type (color legend shared with panel F). (**H**) The STARTRAC-dist index of each cluster in tumor split by treatment status, in which Ro/e denoted the ratio of observed to expected cell number. T_B, tumor tissues in naïve samples; T_A, tumor tissues in treated samples. (**I**) Comparison of selected macrophage clusters in 6 paired naïve and treated tumor samples by Wilcoxon matched-paired signed rank test, * *P* < 0.05. (**J**) Comparison of macrophage cluster percent in naïve and treated tumor samples by Mann-Whitney U test, **** *P* < 0.0001. (**K**) The STARTRAC-dist index of each cluster in treated split by pathological response. Source data are provided as a Source Data file. (**L**) Kaplan-Meier survival curves of MT1G TAM gene signature in ESCA of TCGA dataset

To unbiasedly dissect the cell identities within the TME, we assessed the proportions of fibroblasts, endothelial cells, and immune cell types and further classified them into subsets after quality control (Fig.S2), using well-reported lineage markers (Fig. 1E). Mononuclear phagocytes (MP), which include monocytes, macrophages and dendritic cells (DCs), are important components of the TME. DCs play a vital role in modulating the balance of tumor immunity and homeostasis of T cells [23]. We unbiasedly sub-clustered 7,132 DCs into four phenotypes (Fig. 2A-B): migratory DCs (migDC), plasmacytoid DCs (pDC), and conventional DCs type 1 and 2 (cDC1, cDC2). The role of cDC2 cells in ESCC is complex and not fully understood. Our study found that cDC2 in treatment-naïve group was notably reduced in tumor tissues compared to adjacent normal tissues (Fig. 2B) and increased after treatment (Fig. 2C-D). These findings suggest that cDC2 might help activate the immune response and exhibit an anti-tumor role in the ESCC TME, consistent with Lisong Shen’s ESCC scRNA-seq data [24]. However, there was no significant difference in the proportion of cDC2 in treated samples between pCR and non-pCR groups (*P* > 0.05, Mann-Whitney U test, Fig. 2E).

Macrophages, the largest population of mononuclear phagocytes, were further re-clustered into seven subpopulations (Fig. 2F-H and Fig.S3A-B). HLA-DPB1 TAM was enriched for HLA-DPB1, involved in enhancing tumoral antigen presentation and recruiting CD8 + T cells to facilitate antitumor immune responses, thus, characterized as M1 tumor-associated macrophages (M1 TAM) [25]. In contrast, SPP1 TAM had increased expression of SPP1 and MMP9, and the smallest MT1G TAM expressed high levels of metallothioneins, including MT1G, MT1X, and MT1E. Gene Ontology (GO) analysis of these two clusters showed similar enrichment for genes involved in the immunosuppressive role of neutrophil degranulation [26] (Table S2), and therefore they were both identified as M2 TAM [27, 28]. Compared to adjacent normal tissues in naïve samples, HLA-DPB1 TAM was decreased, while MT1G TAM increased significantly in tumor tissues (*P* < 0.01, Mann-Whitney U test, Fig.S3C). There was no significant alteration in macrophage subsets due to the limited number of 5 paired adjacent normal and tumor tissues (*P* > 0.05, Wilcoxon matched-pairs signed-rank test, Fig.S3D). Following neoICT, MT1G TAM markedly decreased, and LYVE1 TAM increased in 6 paired naïve and treated patients (*P* < 0.05, Wilcoxon matched-pairs signed-rank test, Fig. 2I), as well as in unpaired patients (Mann-Whitney U test, Fig. 2J). The proportion of MT1G was higher and LYVE1 lower in the non-pCR than in the pCR group (Fig. 2K). Additionally, the prognostic value of MT1G TAM program was verified in the ESCA using its distinct gene signature (*P =* 5.02e^− 05^, Fig. 2L, Fig.S3). These results indicate that MT1G TAM exert pro-tumoral role in ESCC.

As indicated earlier, the overall proportion of endothelial cells (ECs) within the ESCC TME increased markedly after neoICT (Fig.S1C). We clustered ECs into five programs (Fig.S4A-B), including arterial ECs, venous ECs, lymphatic ECs, capillary ECs, and proliferating ECs. The largest endothelial population, venous ECs, defined by the expression of the atypical chemokine receptor ACKR1, decreased in naïve tumor tissues compared to normal tissues and increased significantly following neoICT (Fig.S4D-E). In contrast, the proportions of capillary ECs and proliferating ECs were markedly reduced in treated versus untreated samples (Fig.S4D-E). Additionally, the proportion of lymphatic ECs expressing PDPN was markedly increased in naïve tumor (Fig.S4D) and its retention in non-pCR group correlated with poor response to neoICT (Fig.S4F).

### Longitudinal scRNA-seq reveals the heterogeneous CAFs landscape of ESCC

**Fig. 3:**
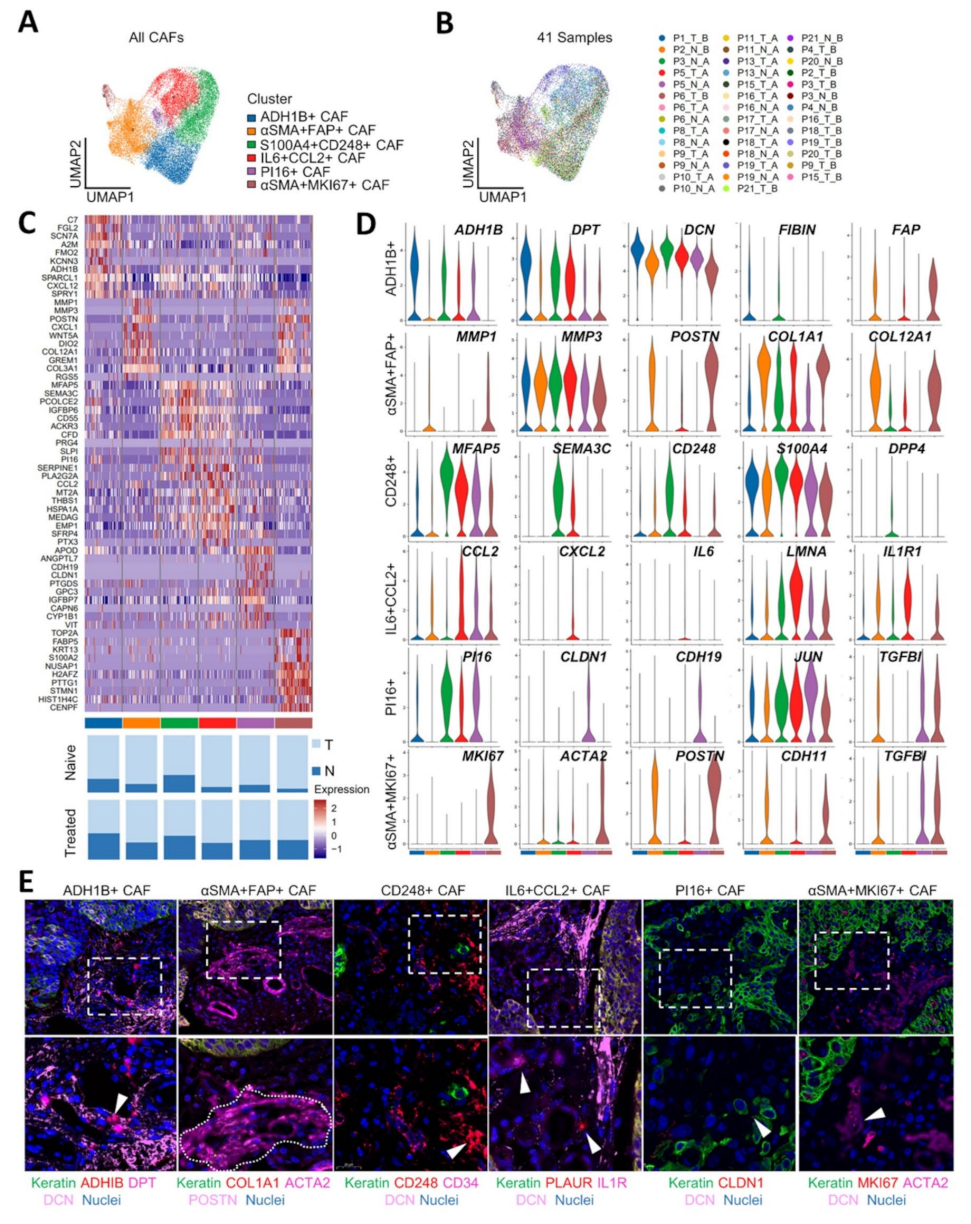
Cancer-associated fibroblast heterogeneity in ESCC. (**A**) UMAP visualization of 6 clusters of 26,962 fibroblast. (**B**) UMAP embedding of single-nucleus profiles in 41 nonmalignant and tumor samples. (**C**) Heatmap showing the expression of top 10 most variable genes across each fibroblast subset (upper panel), color legends as in panel A. The fraction of each fibroblast subset in nonmalignant and tumor samples (lower panel). T, tumor tissue; N, adjacent normal tissue. (**D**) Violin plots showing the expression of selected genes in each fibroblast subset. Color legends as in panel A. (**E**) FFPE ESCC sections were stained for fibroblast markers identified in scRNAseq results. Scale bars = 50 μm (upper), 20 μm (lower). Source data are provided as a Source Data file

Fibroblasts have long been acknowledged as a key stromal cell type in the TME [29]. In 41 samples, we detected 26,962 fibroblast cells and observed significant changes in the proportions of different fibroblast subpopulations within the microenvironment of normal and tumor tissues in naïve and treated group (Fig.S1C and Fig.S5A). To comprehensively decipher the heterogeneous CAF component within ESCC, we clustered CAFs into six subsets via dimensional reduction and unsupervised clustering (Fig. 3A). A UMAP embedding of single-cell profiles for different CAF subsets, colored by the patient, was also conducted (Fig. 3B). Further dissection of CAF clusters revealed distinct transcriptional profiles (Fig. 3C, upper panel). All CAF clusters had a profound abundance in tumor tissues relative to adjacent normal tissues (Fig. 3C, lower panel). To further analyze the transcriptome heterogeneity between distinct populations, a volcano plot showed the top five most differentially active proteins in each cluster (Fig.S5B), and concomitant GO analyses were presented to help define the biological function of each CAF cluster (Fig.S5C). Based on this scRNA-seq analysis, we also identified genes associated with each cluster by multiplexed IHC.

Cluster 1 displayed strong expression of the broad adjacent normal tissue fibroblast marker ADH1B, referred to as ADH1B + CAF [14, 19], and also expressed DPT, which represents a stem-like phenotype in steady-state tissue [22] (Fig. 3C-E). Cluster 5, denoted as PI16 + CAF, was identified as resident fibroblasts with no outstanding fibroblast activation-related gene expression but with intermediate levels of PI16 (Fig. 3C-E). Both clusters 1 and 5 expressed lower levels of canonical ECM components and inflammatory signatures, significantly distinguishing them from mr-CAF and im-CAF. Thus, we termed clusters 1 and 5 as ssl-CAF.

Clusters 2, 3, and 6 demonstrated markedly elevated mechanosensitive signaling mediators and ECM components expression. Cluster 2 notably expressed TGF-β and multiple collagens, including COL12A1, COL3A1, COL1A1, and POSTN [30] (Fig. 3C-E). Meanwhile, cluster 2 demonstrated markedly high expression of αSMA (ACTA2) and FAP, characterized as αSMA + FAP + mr-CAF. Cluster 2 was also enriched with Wnt signaling (e.g. WTN5A, RUNX1, and RUNX2) (Table S4) [31, 32], involving in promoting myofibroblastic CAF differentiation. High levels of ACTA2 and POSTN, along with high expression of MKI67 and NUSAP1, were observed in Cluster 6 [17] (Fig. 3C-E). Thus, we also identified cluster 6 as αSMA + MKI67 + mr-CAF. GO analysis showed that cluster 3 correlated highly with ECM organization (Fig.S5C). However, cluster 3 showed different ECM expression programs, lacked collagen-associated genes and POSTN, but specifically expressed CD248 (TEM1, endosialin), a receptor for collagen types I and IV and fibronectin [33]. Cluster 3 also demonstrated notably elevated expression of mechanoresponsive genes like S100A10, S100A4 (FSP-1), MFAP5, and focal-adhesion-related genes such as SFRP1, RHOA, ITGA11, and ITGBl1 (Table S4) [17]. Therefore, it was designated as CD248 + mr-CAF. Notably, cluster 3 also expressed high levels of angiogenic genes like SEMA3C, SEMA3E, CD34, and VEGFD (FIGF), which might be involved in mediating tumoral angiogenesis (Fig. 3C-E, table S4). We considered the above three CAF clusters as distinct mechanoresponsive phenotypes due to their similar capabilities for mediating ECM biological behaviors, meanwhile, highlighting their unique functions, respectively.

Cluster 4 was enriched in high-level of inflammatory-related genes, including IL6, IL1R1, ICAM1, PLUAR, and chemokines such as CCL2, CXCL14, and CXCL1, but had low level of αSMA and FAP [15]. Additionally, cluster 4 strongly expressed complement factor D (CFD), matrix proteins such as lamin A/C (LMNA), and hyaluronan synthases 1/2 (HAS1/HAS2), which have been identified as new markers for inflammatory CAF [13, 34]( Fig. 3C-E, table S4). We, therefore, refer to this group as im-CAF.

### Differential neoadjuvant therapy responses are associated with heterogeneous CAF subpopulations

**Fig. 4:**
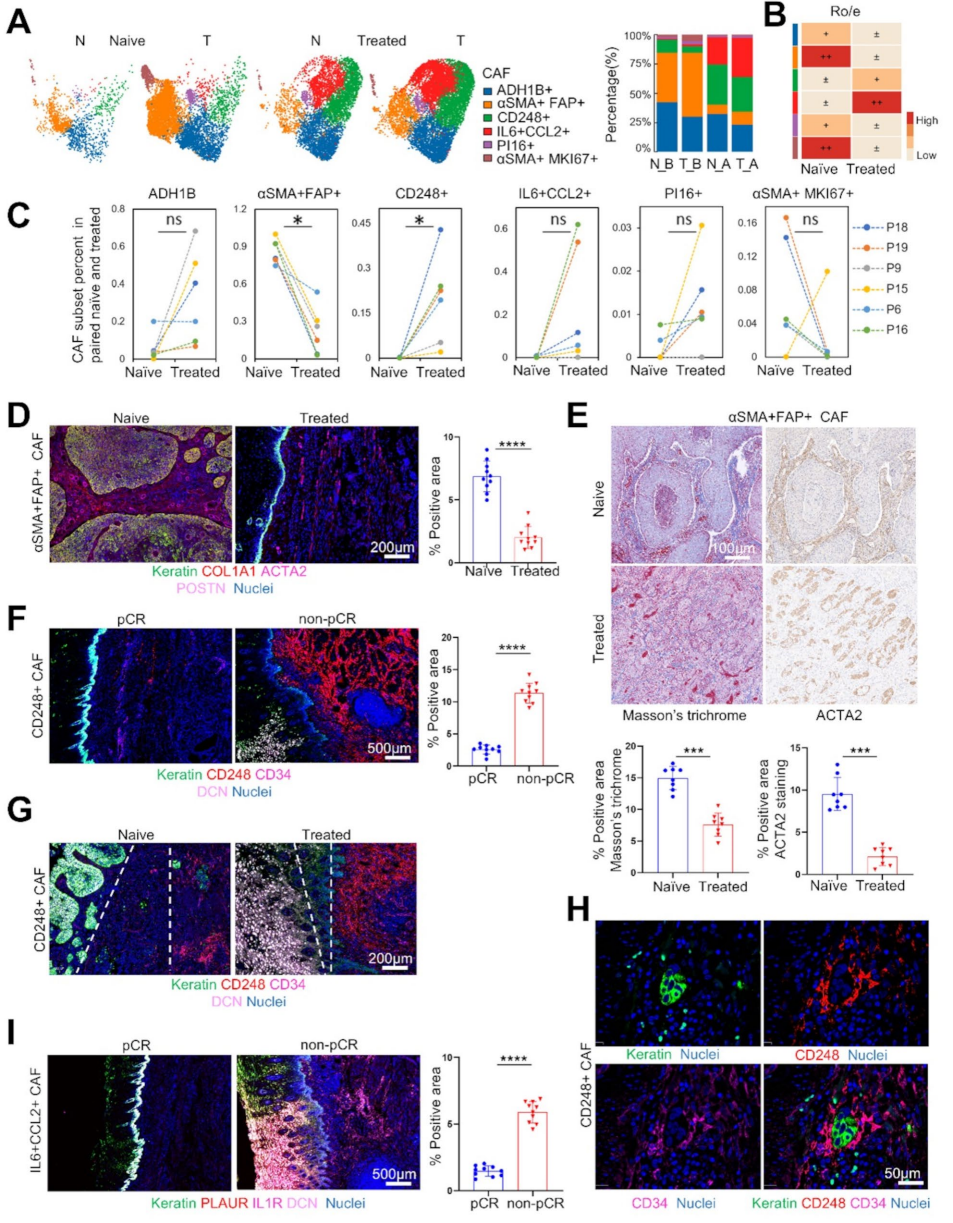
NeoICT modulation of CAF contexture within the tumor microenvironment of ESCC. (**A**) UMAP embedding (left) and the proportion (right) of 6 CAF clusters stratified by treatment and tissue type. N_B, normal tissues in treatment-naïve samples; T_B, tumor tissues in naïve samples; N_A, normal tissues in treated samples; T_A, tumor tissues in treated samples. (**B**) CAF clusters distribution in total naïve and treated tumor specimens estimated by the STARTRAC-dist index, cluster color legend shared with panel A. (**C**) Comparison of 6 CAF clusters population in 6 paired naïve and treated patients. *P* values determined by Wilcoxon signed-rank test, *P < 0.05; ns, no significance. (**D**) Representative images and quantification of mfIHC staining of αSMA + FAP + CAF in pre- and post-neoICT. Scale bars = 200 μm, *n* = 10, ****, *P* < 0.0001, Mann-Whitney U test. (**E**) Representative images of Masson’s trichrome and ACTA2 staining of αSMA + FAP + CAF in pre- and post-neoICT. Scale bars = 100 μm. (**F**) Representative images and quantification of mfIHC staining of CD248 + CAF in pCR and non-pCR. Scale bars = 500 μm, *n* = 10, ****, *P* < 0.0001, Mann-Whitney U test. (**G**) Representative images and quantification of distance between CD248 + CAF and tumor cell. Scale bars = 200 μm. (**H**) Representative images showing the protective niche formation of CD248 + CAF for tumor cell. Scale bars = 50 μm. (**I**) Representative images and quantification of mfIHC staining IL6 + CCL2 + CAF in pCR and non-pCR treated group. Scale bars = 500 μm, *n* = 10, ****, *P* < 0.0001, Mann-Whitney U test. Source data are provided as a Source Data file

To further assess the clinical response of heterogeneous CAF subsets to neoadjuvant anti-PD1 combination therapy in ESCC, we conducted a comprehensive UMAP analysis and compared CAF subclusters in treatment-naïve and treated samples (Fig. 4A). Our STARTRAC-dist index analysis revealed distinct patterns among different CAF subtypes in both naïve and treated samples. CD248 + mr-CAF and IL6 + CCL2 + im-CAF were predominantly enriched after neoICT, contrasting with αSMA + FAP + and αSMA + MKI67 + mr-CAFs, which were enriched in naïve samples (Fig.S5D). No notable alterations were observed in the contribution of ADH1B + and PI16 + CAF subtypes between unpaired naïve and treated samples (Fig. 4B).

In six paired naïve and treated patients, the proportion of αSMA + FAP + mr-CAFs significantly decreased. At the same time, CD248 + mr-CAFs increased in treated tumor tissues (*P* < 0.05 by Wilcoxon signed-rank test, Fig. 4C). mfIHC staining of ESCC FFPE samples confirmed the significant decrease of αSMA + FAP + mr-CAFs in treated versus naïve samples (*P* < 0.0001 by Mann-Whitney U test, Fig. 4D). Additionally, the ECM component derived from αSMA + FAP + mr-CAFs was verified to be notably decreased, with a low density of fiber deposition following treatment, as evidenced by Masson’s trichrome and α-SMA staining (*P* < 0.001 by Mann-Whitney U test, Fig. 4E).

To further understand the transcriptional programs behind αSMA + FAP + mr-CAFs and their relationship to the prognosis of cancer patients, we identified gene signatures with distinct expression patterns across αSMA + FAP + mr-CAFs by Hotspot analysis (Table S5). We scored multiple cancer types from The Cancer Genome Atlas (TCGA) with these signatures based on their expression of αSMA + FAP + mr-CAF genes. This revealed that αSMA + FAP + mr-CAFs correlated with poor prognosis in multiple cancer types, including BLCA (*P* = 0.03), KIRC (*P* = 0), PAAD (*P* = 0.002), LUAD (*P* = 0.022), LUSC (*P* = 0.045) and a poor tendency in ESCA (*P* = 0.123) (Fig.S5E).

Expression of the CAF program in treated group was closely associated with pathological response. A significant increase in CD248 + mr-CAF and IL6 + CCL2 + im-CAF was observed in mfIHC staining following neoICT (*P* < 0.0001 by Mann-Whitney U test, Fig. 4F and I). Despite the lack of collagen expression, CD248 + mr-CAFs exhibited significantly high levels of glycoproteins, such as EMILIN2, FIBULIN1 (FBN1), and FIBRILLIN2 (FBLN2) [35] (Table S4), responsible for focal adhesion, tissue stiffness, mineralization, and mechanical strength. We observed that CD248 + mr-CAFs were far from the tumor cells in naïve tissues but lined the tumor nests in treated tumor tissues, providing a physical barrier to protect malignant tumor cells from immune infiltration and drug delivery (Fig. 4G). Additionally, the high density of CD248 + mr-CAFs formed a protective niche for tumor cells (Fig. 4H). Moreover, the distribution of CD248 + mr-CAFs in non-pCR tissues was notably abundant relative to pCR groups (Fig.S5F). These morphological changes and differential distribution proportion in CD248 + mr-CAFs are attributed to neoadjuvant therapy resistance. CD248, specifically expressed in fibroblasts and abundant in CD248 + mr-CAFs (Fig.S5G, Fig. 3D). In our previous study, we found that CD248 overexpression correlated with an immunosuppressive TME in renal cell carcinoma (RCC), predicting poor prognosis for patients with RCC [36]. In addition, CD248 + mr-CAFs also expressed high levels of angiogenic mediators, participating in angiogenesis, consistent with prior studies that overexpression of CD248 promoted angiogenesis in lung cancer [37] and urothelial carcinoma of the bladder [38]. IL6 + CCL2 + im-CAFs in our study increased significantly in treated group (Fig. 4B), and demonstrated high expression of immunosuppressive inflammatory chemokines and cytokines, such as CCL2, CXCL14, and IL6 (Table S4), correlated with a minor response to neoadjuvant therapy (Fig. 4I), consistent with previous research [39, 40].

### Neoadjuvant combination therapy contributes to the shifts between CAF subpopulations

**Fig. 5:**
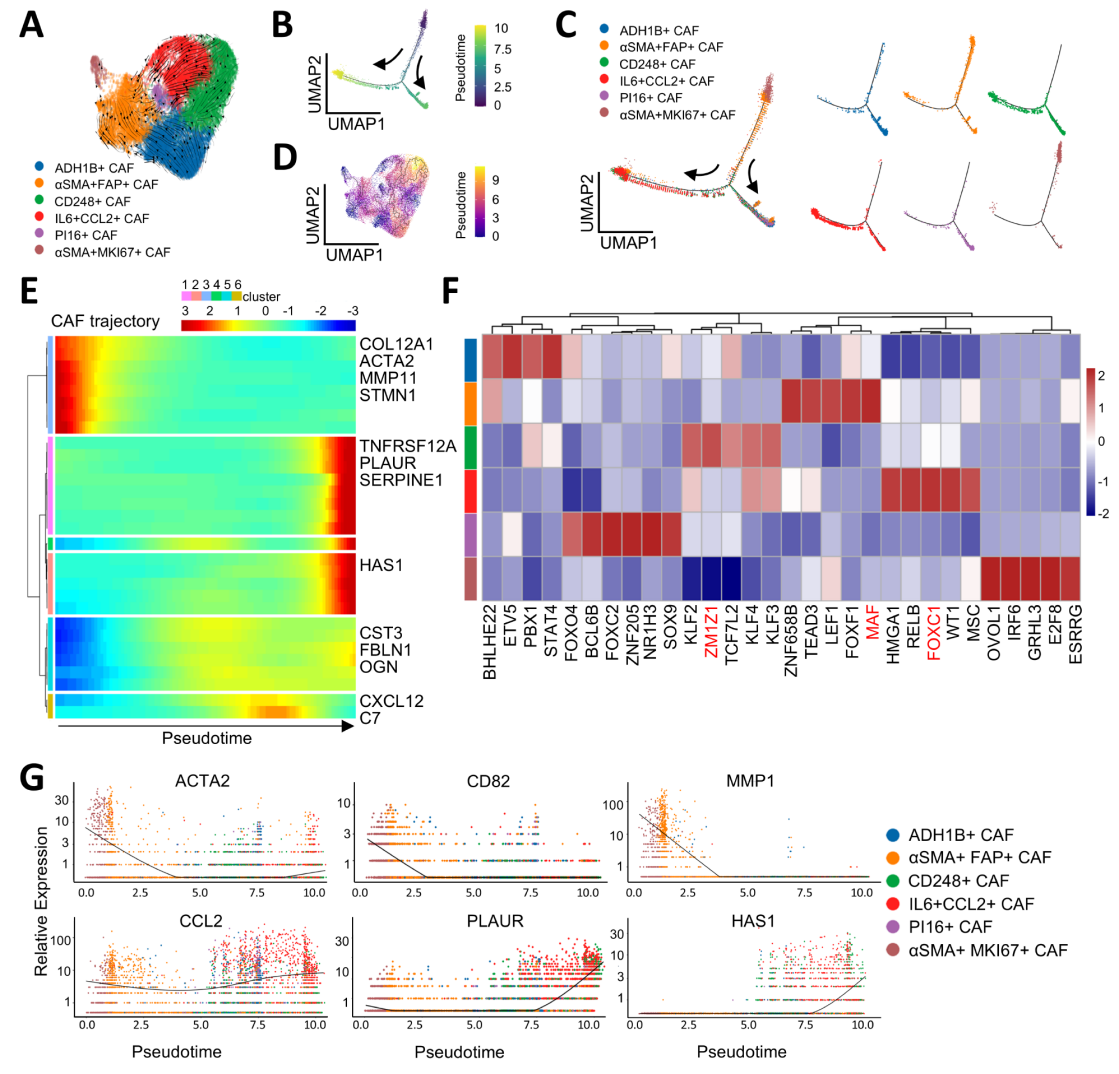
Trajectory analysis of CAF. (**A**) Grid visualization for RNA velocity analysis of CAF subtypes. (**B**) Pseudotime trajectories (Monocle2) for CAF, showing two trajectories. (**C**) Pseudotime trajectories for each CAF. (**D**) Visualization of Monocle3 analysis of CAF subtypes. (**E**) Gene expression dynamics along the CAF trajectory. Genes cluster into six gene sets, each characterized by specific expression profiles, as depicted by a selection of marker genes characteristic for each cluster. (**F**) Heatmap showing the relative expression (z-score) of top five transcription factor in each CAF cluster. Color as in (A). (**G**) A scatter distribution plot showing expression levels of CAF cluster marker genes in pseudotiome order. The color of the dots shows the cluster type of CAF

To further elucidate the underlying mechanisms responsible for the differential responses of CAF clusters to treatment, we explored the cellular trajectories and pseudo-time analysis of six CAF sub-populations. Initially, we designated αSMA + MKI67 + and αSMA + FAP + mr-CAFs as the origins of CAF differentiation based on the results of RNA velocity analysis (Fig. 5A) and SLICE analysis (Table S6) [41]. Pseudo-time analysis using Monocle2 (Fig. 5B-C) and Monocle3 (Fig. 5D) showed that αSMA + FAP + and αSMA + MKI67 + mr-CAFs gradually differentiate into CD248 + mr-CAFs and IL6 + CCL2 + im-CAF.

We identified six sets of differentially expressed genes (DEGs) along the CAF trajectory (Fig. 5E). The first set, consisting of αSMA + FAP + mr-CAF markers (COL12A1, ACTA2 and MMP11) and αSMA + MKI67 + mr-CAF markers (STMN1 and ACTA2), dramatically decreased along the trajectory. In contrast, inflammatory related genes increased significantly in three sets featuring IL6 + CCL2 + im-CAF markers (SERPINE1, TNFRSF12A, PLAUR, and HAS1). Two other sets, including ADH1B + ssl-CAF markers (C7 and CXCL12) and CD248 + mr-CAF markers (FBLN1, CST3, and OGN), increased halfway through the trajectory but then decreased.

A complex network of transcription factors (TFs) orchestrates the differentiation of CAF clusters. We assessed the top five specifically expressed TFs and found that MAF, a critical mediator for promoting immunosuppressive macrophage polarization [42], had the highest expression in αSMA + FAP + mr-CAFs (Fig. 5E). We also observed that ZMIZ1 had the highest expression in the regulatory network of CD248 + mr-CAFs (Fig. 5F). ZMIZ1, a novel HIF-1α co-activator, has been shown to promote metastasis in cancer [43]. FOXC1, another TF promoting invasion and metastasis, was also highly expressed in IL6 + CCL2 + im-CAFs (Fig. 5F). These findings suggest that these above three CAF clusters might be associated with poor prognosis in ESCC.

The expression of ECM-associated genes such as COL12A1, ACTA2, and MMP11 was significantly downregulated, concomitant with the decrease of αSMA + MKI67 + and αSMA + FAP + mr-CAFs (Fig. 5G). Meanwhile, inflammatory-associated mediators like HAS1, CCL2, and PLAUR were gradually upregulated, accompanying the increase of IL6 + CCL2 + im-CAFs (Fig. 5G).

### Neoadjuvant combination therapy increases CD8 + T cell infiltration but promotes their exhaustion

**Fig. 6:**
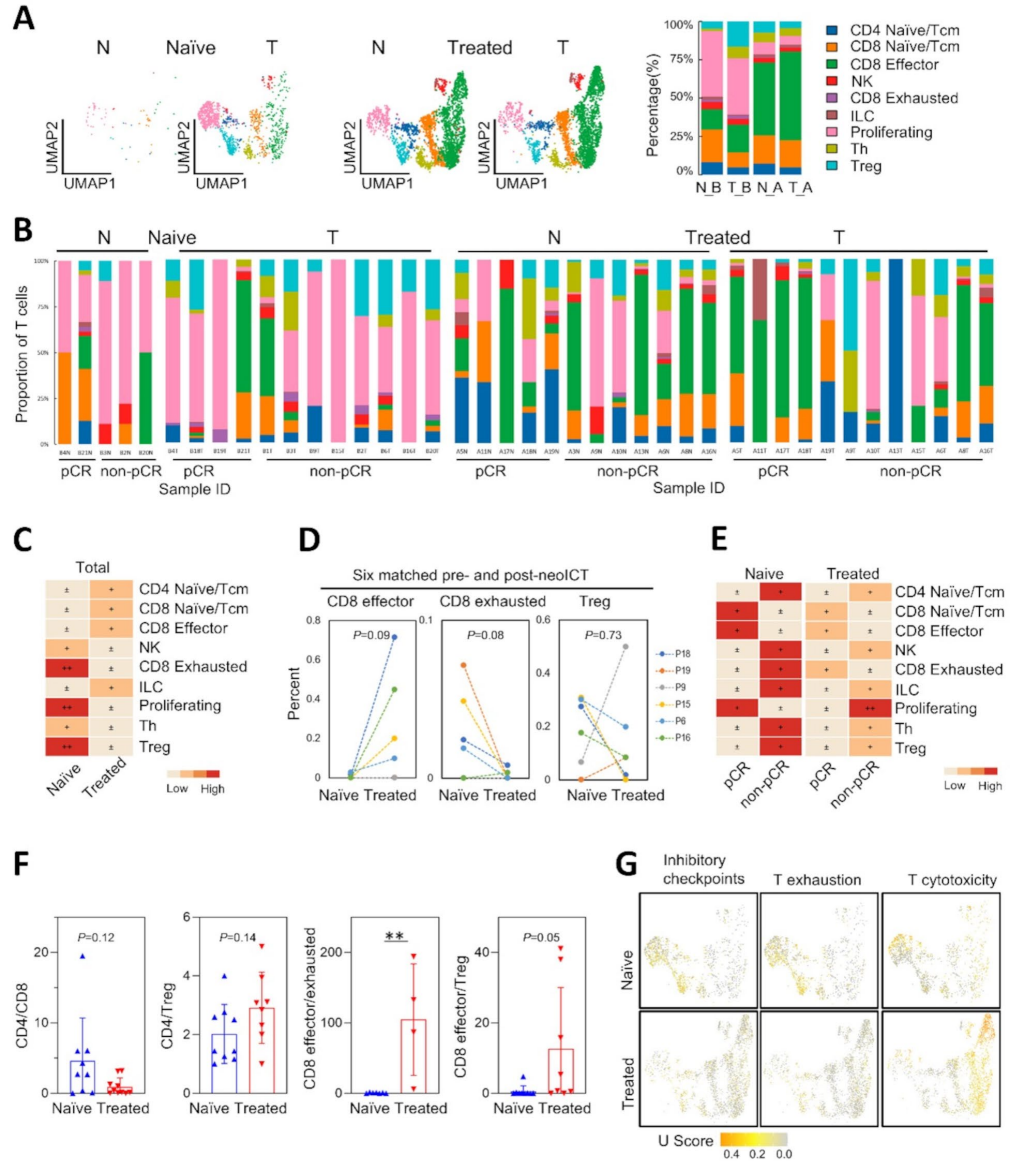
Characterization of CD8 + T cells in pre- and post-neoIRT ESCC. (**A**) UMAP embeddings (left) and proportion comparison (right) of T clusters stratified by treatment and tissue type. N_B, normal tissues in treatment-naïve samples; T_B, tumor tissues in naïve samples; N_A, normal tissues in treated samples; T_A, tumor tissues in treated samples. Tcm, the central memory; ILC, innate lymphoid cell. (**B**) T cell-type distributions stratified by treatment, tissue type and pathological response across 41 samples. Proportions (y axis) of cell subsets (color legend, shared with panel A) across naive (*n* = 17) (left) versus treated (*n* = 24) (right). (**C**) Heatmap of STARTRAC-dist index of each T cluster split by treatment status. (**D**) Comparison of seleted T clusters in 6 paired naïve and treated tumor samples by Wilcoxon matched-paired signed rank test, * *P* < 0.05. (**E**) Heatmap of STARTRAC-dist index of each T cluster split by treatment status and pathological response. (**F**) CD4:CD8, CD4:Treg, CD8 effector: exhausted and CD8 effector: Treg ratios stratified by treatment status. *P* values determined by Mann-Whitney test. (**G**) UMAP embeddings overlaid with selected signature module scores and distributions of module scores stratified by chemotherapy treatment. Source data are provided as a Source Data file

T cells are the most abundant and highly heterogeneous among tumor-infiltrating lymphocytes (TILs) in TME. Identifying the immune status of both naïve and treated tumor samples is possible through the dissection of T cell populations. We clustered 7,911 T cells and identified natural killer (NK) cells and 8 distinct T cell subtypes using established markers (Fig. 6A-B and Fig.S6A). The CD4 naïve/central memory subset was characterized by high expression of IL7R, TCF7, SELL, and CCR7 genes. FOXP3-expressing regulatory T cells (Tregs) showed high IL2RA and IKZF2 expression levels. The CD8 naïve/central memory subset exhibited the memory marker IL7R, while NKG7, GZMB, and GNLY characterized the CD8 effector T subset. This subset also expressed low levels of LAG3, indicating that it included pre-exhausted CD8 T cells [44]. CD8 exhausted T cells were identified based on the expression of immune checkpoints (LAG3, HAVCR2, CTLA4). The remaining clusters included NK cells, innate lymphoid cells (ILCs), and proliferating T cells.

Following anti-PD1 combination therapy, the populations of proliferating T cells, CD8 exhausted T cells, and Tregs significantly decreased, whereas CD8 effector T cells increased (Fig. 6C-E, Fig.S6B). In naïve samples from the pCR group, we found higher levels of CD8 effector T cells, lower levels of CD4 naïve T and Tregs than the non-pCR group (Fig. 6E), elucidating a relatively “hot” immune status conducive to neoICT. In treated samples from the pCR group, CD4 naïve T and Tregs were also lower compared to non-pCR group. The abundance of CD4 naïve T cells and Tregs is closely correlated, both indicating poor prognosis for cancer patients [45], which might contribute to resistance to neoICT in non-pCR group. However, pCR group were also accompanied by higher levels of exhausted T cells (Fig. 6E). Overall, neoICT, on the one hand, enhance the function of cytotoxic T cells while also accelerate the exhaustion of CD8  T cells, accounting for the consistent increase in the proportion of CD8 effector and exhausted T cells in the pCR-treated group.

The CD4:CD8 ratio was 2.72 (median) in naïve tumor tissue but fell to 0.26 following anti-PD1 combination therapy (Fig. 6F). Moreover, neoICT markedly reduced the proportion of Tregs and exhausted T cells within the tumor. The CD4:Treg ratio increased more than 2-fold, from 1.44 to 2.96 (*P* = 0.14); the CD8 effector: Treg ratio increased from 0.06 to 2.98 (*P* = 0.05); and the CD8 effector: exhausted T ratio notably increased from 0.67 to 109.60 (*P* = 0.006) (Fig. 6F). Furthermore, we used the enrichment of selected hallmark gene sets to estimate the signature score distributions of T cell subsets. Consistent with changes in the proportions of T cell subsets, inhibitory checkpoint expression on Tregs was downregulated, while the cytotoxic function of CD8 effector T cells and NK cells was notably upregulated following anti-PD1 combination therapy (Fig. 6G). Overall, the neoadjuvant therapy remodeled the immunosuppressive TME, shifting it from a “cold” to a “hot” immune landscape.

### Crosstalk between CAFs and immunosuppressive T cells lead to the drug resistance of ESCC

**Fig. 7:**
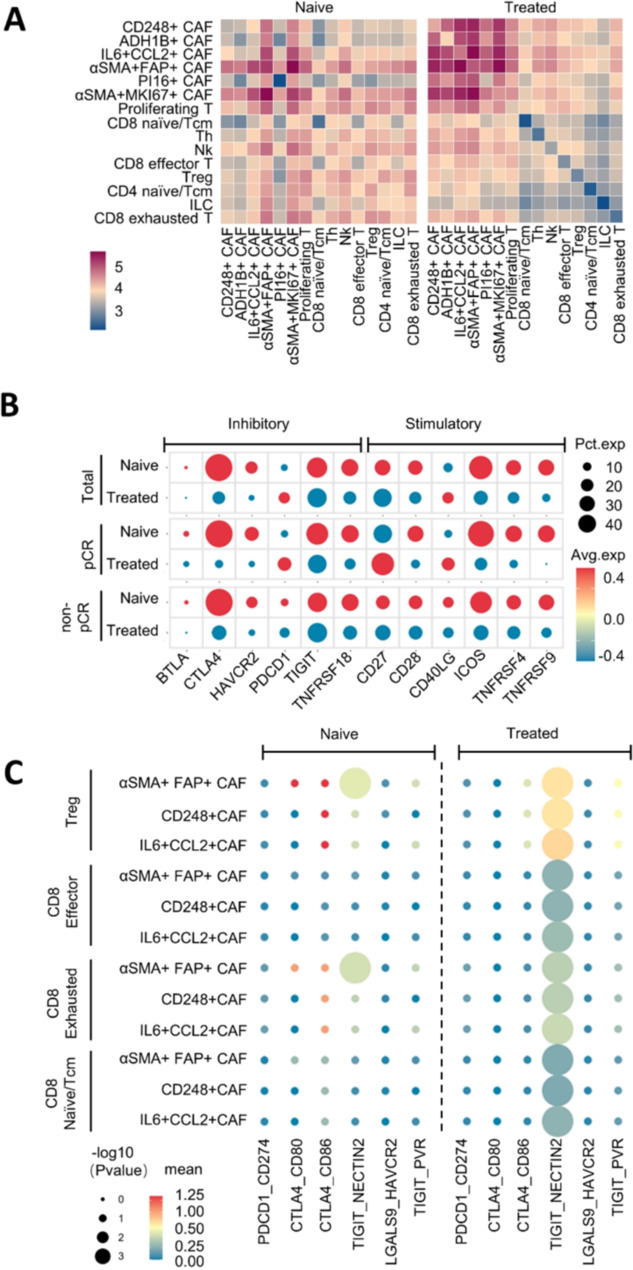
Characterization of checkpoint molecules and ligand-receptor interactions in naïve and treated group in ESCC. (**A**) Clustered heatmap showing two-sided Spearman correlation coefficients between all CAF clusters and T clusters in tumor samples. Left: untreated samples, right: treated samples. (**B**) Differential gene expression of inhibitory and stimulatory checkpoint molecules in CD8 + T cells between treated and untreated samples. (**C**) Dotplots of CellphoneDB output (see Methods) showing significance (-log10 *P* value) and strength (mean value) of checkpoint molecule ligand-receptor interactions between CAF and T cells comparing untreated and treated samples. Source data are provided as a Source Data file

Numerous studies have highlighted the roles of CAFs in modulating T cell activities and functions. To investigate alterations in cell-cell communication within the TME of ESCC before and after neoadjuvant therapy, we utilized CellPhoneDB [46]. We identified recruitment-associated ligand-receptor pairs between CAFs and T cells (Fig. 7A, left panel) and observed a general increase in cell-cell interactions in treated samples (Fig. 7a, right panel). Given that combination therapy with a PD1 inhibitor and chemotherapy has shown benefits in ESCC clinical trials, we further analyzed treatment-related checkpoint molecule expression in T cells. Among inhibitory checkpoint molecules in naïve samples, CTLA4 showed the highest and broadest expression, followed by TIGIT, while PDCD1 expression was extremely low (Fig. 7B). We also evaluated the relationship between pathological response and checkpoint molecule expression. In naïve samples, PD1 expression was lower in the pCR group than in the non-pCR group (Fig. 7B), suggesting that the non-pCR naïve group had a “colder” tumor immune environment, which accounted for their relatively low response to combination therapy. PD1 is not expressed in naïve T cells but is induced in activated T cells [47]. After treatment, co-inhibitory checkpoints, including PD1, were upregulated, especially in the pCR group but not in the non-pCR group, consistent with the findings of Liu et al. study [48]. This indicates that T cells were substantially activated in the pCR group, whereas they remained naïve in the non-pCR group. On the other hand, we found that in the pCR group, stimulatory molecules CD27 and CD40L were upregulated, enhancing the anti-tumor activities of T cells (Fig. 7B). It has been demonstrated that intermediately activated/exhausted CD8 + T cells co-express both T cell activation and exhaustion gene signatures following PD-1-based therapies [49].

We observed a high level of CTLA4-CD86/80 interaction between CD248 +, IL6 + CCL2 + CAFs and Tregs, exhausted T cells in naïve samples (Fig. 7C). Interestingly, following treatment, CTLA4-CD86/80 crosstalk decreased. At the same time, a strong co-inhibitory TIGIT-NECTIN2 signal emerged between CD248 +, IL6 + CCL2 + CAFs and T cells (Fig. 7C). Nectin Cell Adhesion Molecule 2 (NECTIN2, PVRL2/CD112), a member of the Nectin-like molecule family, affects the function of cytotoxic T lymphocytes and inhibits anti-tumor immunity. We found that NECTIN2 expression in CD248 + and IL6 + CCL2 + CAFs was significantly upregulated in treated group compared to the naïve samples (Fig.S7A). Additionally, the co-inhibitory TIGIT-NECTIN2 interactions between Tregs and CAFs were substantially higher in non-pCR than pCR group. Conversely, the TIGIT-NECTIN2 interactions between exhausted CD8 + T and CAFs were relatively lower in non-pCR group (Fig.S7B). These interactions difference was in concordant with the pattern of infiltration of Treg and decrease of exhausted CD8 + T in non-pCR group (Fig. 6E). The key PDCD1/CD274 interaction was barely observed in both naïve and treated samples (Fig. 7C), whereas, moderately upregulated between exhausted CD8 + T and CAFs in non-pCR group (Fig. S7B). In addition, the CTLA4-CD86 and TIGIT-PVR interactions between Tregs and CAFs were substantially higher in non-pCR group than pCR group (Fig. S7B). Overall, these data depict an interactive immune environment following neoICT, and indicate that the TIGIT-NECTIN2 pair serves as the key ligand-receptor interaction between CD248 +/IL6 + CCL2 + CAFs and Tregs, potentially contributing to drug resistance in neoadjuvant combination therapy.

## Discussion

Clinical studies have demonstrated that immune checkpoint inhibitors combined with chemotherapy can enhance the host’s immunity and inhibit tumor immune evasion. Dynamic remodeling of the TME plays a crucial role in the response to treatment based on the differences in infiltrating cell types between tumor and adjacent normal tissues. In this work, we uncovered the heterogeneity of major TME components and their contributions to the resistance of neoadjuvant therapy in ESCC. Firstly, we found that mononuclear phagocytes infiltration increased following anti-PD1 combination therapy. cDC2 levels reduced sharply in the TME of the naïve group relative to adjacent normal tissues but increased markedly in the treated group. Generally, although DCs are a minority in tumors, cDC2 cells specialize in antigen presentation and T-cell activation, which is crucial for recognizing and killing cancer cells. cDC2 plays a dual role in tumor immunity, and multiple studies demonstrate that the expression of cDC2 gene markers is associated with a positive prognosis in human and mouse cancer models [50]. Our work also showed that cDC2 was suppressed in ESCC and activated by anti-PD1 combination therapy, leading to better outcomes. Consistent with previous reports on subtype heterogeneity of macrophages, we identically identified an SPP1 + macrophage phenotype [51], a M2 TAM population, and also identified a novel but minor M2 TAM phenotype, MT1G + TAM. SPP1 + and MT1G + were both abundant in non-pCR treated group than pCR group, indicating this phenotypic macrophage might serve as minor response predictive of neoadjuvant combination therapy.

CAFs, abundant and heterogeneous stomal cells in TME, are critically involved in cancer progression and drug resistance. Here, we provided an overview of the CAF compartments at the single-cell level and spatial location, and showed that CAF populations were enriched in almost all post-treatment groups. We identified six CAF subsets: three mr-CAF subsets, one im-CAF subset, and two ssl-CAF subsets. Our data did not disclose an antigen-presenting CAF phenotype, consistent with other scRNA analyses of ESCC [52, 53]. αSMA + FAP + mr-CAFs are highly enriched in both ECM-associated genes (COL1A1, MMP3, POSTN) and the contractile protein αSMA, typical of myofibroblastic CAFs. αSMA + FAP + mr-CAFs predominated in tumor tissue relative to adjacent normal tissue in patients with treatment-naïve ESCC. We also found that high enrichment of αSMA + FAP + mr-CAFs predicted poor prognosis through pan-cancer analysis using TCGA datasets. ESCA in TCGA includes esophageal adenocarcinoma and ESCC, accounting for no significant correlation between αSMA + FAP + mr-CAF and ESCA. Generally, αSMA + FAP + mr-CAFs express high collagen levels and function as a barrier to effective drug delivery to cancer cells. At the same time, ECM degradation after neoadjuvant combination therapy can improve T cell infiltration [54].

Using trajectory inference, we observed CD248 + mr-CAF and IL6 + CCL2 + im-CAF were both significantly enriched following neoadjuvant combination therapy. Intriguingly, the ECM program of CD248 + mr-CAF was distinct from that of αSMA + FAP + mr-CAF. Specifically, CD248 + mr-CAF expressed low levels of fibrillar collagen but high glycoproteins associated with extracellular matrix organization and angiogenic mediators. We observed high density of CD248 + mr-CAFs formed a survival niche for tumor cells to escape the anti-tumor effect of neo-ICT, consistent with the recent research that indicates ECM-CAF with high CD248 expression shape an immunosuppressive tumor microenvironment by forming a stromal barrier to inhibit T-cell function [55]. CD248, a novel specific marker of myofibroblasts and tumor vessel-associated mural cells [56], which functions in cell adhesion, migration, and angiogenesis in multiple types of cancer, including lung cancer, melanoma, and renal cell carcinoma [36, 37, 57, 58]. Furthermore, it is noteworthy that CD248 + mr-CAFs not only possess the ability to regulate ECM organization and facilitate angiogenesis but also exhibit a significant upregulation of PI16, DPP4, and CD34, suggesting a resemblance to a stable state-like molecular profile [19]. However, according to the findings of Buechler et al., PI16 and CD34 were most enriched in a human perturbed-state fibroblast atlas across multiple tissues, and PI16 and DPP4 were most enriched in perturbed mouse tissues [22]. Thus, regarding of the universal signature of PI16, we classified CD248 + mr-CAF as mechanoresponsive CAF rather than steady state-like CAF. A recent study demonstrated that CAFs expressed high levels of PI16 and CD34 localized to the blood vessel adventitia in lung cancer [14]. Therefore, in our work, multi-dimensional evidence proved that CD248 + mr-CAFs are associated with angiogenesis, a hallmark of tumor malignancy in ESCC.

It is widely known that inflammatory CAFs with pro-inflammatory cytokines lead to therapy resistance in multiple cancers, such as pancreatic carcinoma, breast and colorectal cancer [12, 13]. In our findings, IL6 + CCL2 + im-CAF expressed high levels of CCL2, CXCL14, IL6, and PLAUR. CCL2 and CXCL14, potent inducers of CAF pro-tumorigenic functions, enhance macrophage infiltration [59, 60]. Another study also shows that CXCL14-expressing CAFs promote breast cancer epithelial-to-mesenchymal transition, invasion, and metastasis by interacting with the ACKR2 receptor [61]. Compelling evidence suggests that IL-6 derived from CAFs mediates therapeutic resistance, including chemoresistance by upregulating CXCR7 expression via STAT3/NF-κb pathway in ESCC [62], and IL-6 blockade improves checkpoint blockade therapy and promotes tumor immunity [63]. PLAUR, the receptor of PLAU, is known as the urokinase plasminogen activator receptor (uPAR). PLAU is overexpressed in various cancers, including ESCC, and tumor-secreted PLAU promotes the formation of iCAF via the uPAR/AKT/NF-κB/IL8 pathway [64]. Interestingly, our data showed that IL6 + CCL2 + im-CAFs have high expression of PLAUR. Further validation is needed to understand the underlying interactions between PLAU-positive ESCC tumor cells and PLAUR-positive iCAFs.

The intricate composition of, and communication between, cells in the TME contribute to the complexity of neoICT. Our work also investigates the relative importance of immune checkpoint molecules in tumor-infiltrating T cells and the ligand-receptor interactions between CAFs and T cells in different pathological responses. In our analysis, the CTLA4-CD80/86 axis was the key inhibitory checkpoint component in treatment-naïve patients, whereas PD1 was barely expressed in CD8 + T cells. This might explain the limited efficacy (MPR 57.5% [6]) of Tislelizumab combined with chemotherapy. Our findings demonstrate high ligand-receptor interactions between different CAF clusters and tumor-infiltrating T cells in treated samples. Notably, the TIGIT-NECTIN2 co-inhibitory interaction between CD248 +/IL6 + CCL2 + CAFs and Tregs was significantly elevated in non-pCR treated group compared to pCR group. TIGIT is co-inhibitory receptor first reported by Yu et al. in 2009 [65], highly expressed in many malignant tumors, including ESCC, and is closely related to patient prognosis [66]. Research has shown that TIGIT’s expression can more reliably identify exhausted CD8 + T cells than PD-1 [67]. TIGIT binds to two ligands, CD155 and NECTIN2, to regulate the function of T cells and NK cells [68]. Recent research has illustrated that the TIGIT-NECTIN2 pathway drives immune cells toward an immunosuppressive and exhausted state in hepatocellular carcinoma [69] and breast cancer [70]. In our findings, high TIGIT expression and prominent TIGIT-NECTIN2 interactions between Tregs and CAFs work together to reshape the immunosuppressive TME, consistent with recent research [70]. Therefore, targeting TIGIT with monoclonal antibodies might effectively restore and reactivate the function of T cells and NK cells [71]. Taken together, blocking TIGIT may be more effective as a first-line immunotherapy than PD1 inhibitors in ESCC.

There were also some limitations in this study. Although there were eighteen patients enrolled in scRNA-seq analysis, only six patients were paired pre- and post-treatment. The potential reason was that the treatment-naïve samples were obtained from EBUS biopsies. It was difficult to ensure remaining tumor tissues for scRNA-seq, apart from application for pathological diagnosis. Further studies with larger paired samples size would help deciphering the dynamic alteration of TIME landscape in ESCC. Secondly, our work did not compare simultaneously and horizontally with patients with neoadjuvant chemotherapy or neoadjuvant immunotherapy regimen alone. Therefore, in the analysis of immune environment remodeling, we could not distinguish the concrete regulatory effect of chemotherapy or PD-1 inhibitor. Thirdly, due to lack of murine esophageal cancer cell lines, it is difficult to establish an ESCC animal model in immune-intact mice and further validate the regulatory role of CAF in the ESCC TIME.

In summary, our study offers a comprehensive analysis of the cellular composition of human ESCC, the impact of neoadjuvant anti-PD1 combination therapy on the immune landscape, and specifically highlights the phenotypic and functional heterogeneity of CAFs within the TME of ESCC. These discoveries can potentially inform more rational treatment strategies for patients with a low response to neoadjuvant immunochemotherapy for ESCC.

## Methods

### Human tumor specimens

The clinical characteristics of the 18 patients are available in Table S1. 41 specimens were surgically obtained from tumor tissues and adjacent normal tissues before and after treatment (tislelizumab plus carboplatin and nab-paclitaxel, 3 months). 17 specimens from 12 patients were harvested before treatment, while 24 specimens from 13 patients were harvested after treatment. Six patients had matched treatment-naïve and treated specimens. The adjacent normal tissues were obtained at least 5 cm away from the tumor tissues.

### Tissue processing

Fresh tissues were stored in sCelLive™ Tissue Preservation Solution (Singleron) on ice after surgery within 30 min. The specimens were washed three times with Hanks Balanced Salt Solution (HBSS), cut into small pieces with scissors, and enzymatically digested with 3 mL of sCelLive™ Tissue Dissociation Solution (Singleron) using the Singleron PythoN™ Tissue Dissociation System at 37 °C for 15 min. Cell suspensions were filtered through a 70 μm cell strainer and then lysed in GEXSCOPE^®^ RBC lysis buffer (RCLB, Singleron). The mixture (Cell: RCLB = 1:2, volume ratio) was incubated at room temperature for 8 min to remove red blood cells. The mixture was then centrifuged at 300 g at 4 °C for 5 min to remove the supernatant and washed once with PBS. Cells were resuspended in a buffer comprising DPBS with 5% bovine serum albumin (BSA, Yeasen, China) and then counted using a hemocytometer. Cell viability was evaluated microscopically by staining the samples with 0.4% Trypan Blue (Solarbio, China).

### Droplet-based single-cell RNA sequencing

Freshly prepared single-cell suspensions (2 × 10^5^ cells/mL) were processed immediately according to the manufacturer’s protocol from 10x Genomics. Briefly, single-cell suspensions were loaded onto a microwell chip using the Singleron Matrix^®^ Single Cell Processing System. Barcoding beads were subsequently collected from the microwell chip, followed by reverse transcription of the mRNA captured by the barcoding beads to obtain cDNA and PCR amplification. The scRNA-seq libraries were constructed according to the protocol of the GEXSCOPE^®^ Single Cell RNA Library Kits (Singleron), along with the reverse transcription master mix and single-cell 3 gel beads [72]. Individual libraries were diluted to 4 nM, pooled, and sequenced on a NovaSeq 6000 (Illumina, USA) with 150 bp paired-end reads per sample.

### scRNA-seq data analysis

Raw sequencing reads data were processed to generate gene expression profiles using CeleScope v1.1.1 (Singleron Biotechnologies) with default parameters. Barcodes and unique molecular identifiers (UMIs) were extracted from R1 reads and corrected. Adapter sequences and poly-A tails were trimmed from R2 reads, and the trimmed R2 reads were aligned against the GRCh38/hg38 transcriptome using STAR (v2.6.1b). Uniquely mapped reads were then assigned to exons with FeatureCounts (v2.0.1). For further analysis, reads with the same barcode, UMI, and gene were grouped to create a gene expression matrix. The R package Seurat v3.1.2 was used for quality control, dimensionality reduction, and clustering. For each sample dataset, we filtered the expression matrix using the following criteria: (1) cells with gene counts less than 200 or with top 2% gene counts were excluded; (2) cells with top 2% UMI counts were excluded; (3) cells with mitochondrial content > 50% were excluded follow Jeff DeMartino’s mitochondrial filtering criterion [73]; (4) genes expressed in fewer than 5 cells were excluded. After filtering, 232,710 cells were retained for downstream analyses, with an average of 1,343 genes and 4,782 UMIs per cell. Data were normalized and scaled using the NormalizeData and ScaleData functions. FindVariableFeatures selected the top 2,000 most variable genes for principal component analysis (PCA). Cells were separated into 18 clusters by FindClusters, using the top 20 principal components and a resolution parameter set at 0.8. Cell types and clusters were visualized using Uniform Manifold Approximation and Projection (UMAP). Batch effects between samples were removed by Harmony v1.0, using the top 20 principal components from PCA [74].

### Cell type annotation

Using Cell-ID, each cell type and gene signature was recognized and identified [75]. For cluster annotation, the frequency of each cell type was calculated in each cluster, and the cell type with the highest frequency was chosen as the cluster’s identity. A canonical marker expression analysis using SynEcoSys™ (Singleron Biotechnology) was employed to identify the cell types of each cluster. Drawing from CellMakerDB, PanglaoDB, and recently published literature, SynEcoSys™ contains well-defined cell type markers for single-cell sequencing data. The marker genes used for annotation of cell types included EPCAM, Keratin genes (KRT5, KRT14), and SPRR1A for epithelial cells; CD3D, CD3E, and TRAC for T cells; LYZ, CD14, CD68, and MCR1 for mononuclear phagocytes; CD79A/B and MS4A1 for plasma cells; CD79A/B, JCHAIN, and MZB1 for B cells; CLDN5, PECAM1, and CDH5 for endothelial cells; DCN, COL1A2, and COL1A1 for fibroblasts; TAGLN and CNN1 for smooth muscle cells; and TPSAB1 and TPSB2 for mast cells. Further sub-clustering was performed for fibroblasts, immune cells, and other major cell types for in-depth investigation and better annotation of clusters. Cells from specific clusters were extracted and reclustered for more detailed analysis, following the same procedures described above and setting the clustering resolution at 0.8.

### Inferring copy number alterations detection

The InferCNV package was used to detect chromosomal copy number variations, applying parameters recommended for 10× genomics data at each sample level [76]. Non-malignant cells (CAF, endothelial cells, and immune cells) were used as the reference normal sets. The relative expression values were centered at 1, with 1.5 standard deviations from the residual-normalized expression values serving as the floor and ceiling. Heatmaps generated by the R pheatmap function displayed the inferred CNAs on either the short or long arm or the full length of the chromosomes. The CNV score for each cell was calculated as the quadratic sum of the CNA region.

### Differential expression analysis

To identify differentially expressed genes (DEGs), we employed the Seurat FindMarkers() function based on the Wilcoxon rank-sum test with default parameters. We selected genes expressed in more than 10% of the cells in both of the compared groups and with an average log (Fold Change) value greater than 0.25 as DEGs. Statistical significance was evaluated using an adjusted *p*-value below 0.05, calculated by Bonferroni Correction.

### Functional and pathway enrichment analysis

To investigate the potential functions of CAF subclusters, we used the ClusterProfiler R package v 3.16 to analyze Gene Ontology (GO) and Kyoto Encyclopedia of Genes and Genomes (KEGG) data [77]. Significantly enriched pathways with adjusted *p*-values below 0.05 were plotted as bar plots.

### Functional gene module analysis

Hotspot was employed to identify functional gene modules that illustrate heterogeneity within CAF subpopulations [78]. Briefly, we used the ‘danb’ model and selected the top 500 genes with the highest autocorrelation z-scores for module identification. Modules were then identified using the create_modules function with min_gene_threshold = 15 and fdr_threshold = 0.05. Module scores were calculated using the calculate_module_scores function. The related gene set of MT1G TAM is available in Table S3, Supporting Information. The gene signatures for αSMA + FAP + CAF, CD248 + CAF, and IL6 + CCL2 + CAF are available in Table S5.

### Pseudotime trajectory analysis

*Monocle2*: Cell differentiation trajectories of monocyte subtypes were reconstructed usingMonocle2 v 2.10.0 [79]. The top 2,000 highly variable genes were selected by Seurat’s FindVariableFeatures() function for constructing the trajectory, and dimension reduction was performed by DDRTree(). The trajectory was visualized using the plot_cell_trajectory() function in Monocle2. *Monocle3*: Monocle3 v1.0.0 was used to reconstruct cell differentiation trajectories [80]. Differentially expressed genes were used to sort cells in order of spatial-temporal differentiation. We used UMAP to perform graph_test() and dimension reduction, and to recognize the trajectory by the learn_graph() function. Finally, the trajectory was visualized using the plot_cells() function.

### RNA velocity-based cell fate tracing

Using Velocyto (v0.2.3) and scVelo (v0.17.17) in Python with default parameters, we analyzed RNA velocity from a BAM file containing CAFs and the reference genome GRCh38/hg38 [81]. To ensure consistency in visualization, the results from the Seurat clustering analysis were projected onto the UMAP plot.

### STARTRAC-dist analysis

The enrichment of specific cell clusters in tissues was measured using STARTRAC-dist [82]. We first applied a chi-squared test to evaluate whether the distribution of the cell clusters of interest significantly deviated from random expectations. The STARTRAC-dist index is defined as Ro/e, where Ro is the observed cell count and e is the expected cell count. If Ro/e > 1, it suggests that cells from the cluster of interest are observed more frequently than would be expected at random in the specific tissue, indicating enrichment. Conversely, if Ro/e < 1, it suggests that cells from the cluster of interest are observed less frequently than would be expected at random, indicating depletion. The tissue preference of the cell clusters of interest can be efficiently quantified by calculating STARTRAC-dist indices via Ro/e.

### Cell-cell communication analysis

Cell-cell interactions between CAF subtypes and T cells were predicted based on known ligand-receptor pairs using CellphoneDB (v2.1.0) [46]. A total of 1,000 permutations were necessary for generating the null distribution of average ligand-receptor pair expression in randomized cell identities. All other parameters were kept at their default values. Individual ligand or receptor expression was thresholded using average log gene expression distributions across all cell types. In CellphoneDB, interaction pairs with *p*-values < 0.05, and log expressions > 0.1 were considered significant and were visualized using heatmap_plot and dot_plot.

### Survival analysis of CAF gene signatures in TCGA

Multiple bulk RNA-seq transcriptome data and clinic information were obtained from TCGA database. We further plotted the sample-by-sample enrichment of these CAF populations among different TCGA tumor types (BLCA, KIRC, PAAD, LUAD, LUSC and ESCA). To evaluate relationship between the target gene set and clinical factors, ssgsea were used to calculate a score of each gene sets for samples, then samples were assigned into two groups (High group and Low group) using median score as the cutoff. Differences in overall survival and progression-free interval between high and low groups were compared using Kaplan-Meier curves, with *p*-values calculated via log-rank test, using the Survival package in R.

### Multiplex fluorescent immunohistochemistry staining

Human tissue specimens were provided by Tangdu hospital, Air Force Medical University under an approved Institutional Review Board protocol. Multiplex fluorescent immunohistochemistry staining (mfIHC) of 4 μm formalin-fixed, paraffin-embedded (FFPE) sections was performed according to the protocol developed by previous literature [83]. Briefly, slides were baked, deparaffinized and then rehydrated. Citrate buffer (PH 6.0) was used for antigen retrieval at 95 °C for 30 min, followed by incubation in 3% H_2_O_2_ for inactivating endogenous peroxidase for 20 min, then blocked with 10% normal goat serum for blocking nonspecific sites for 30 min. Primary antibodies were sequentially applied at 4 °C overnight, followed by a secondary antibody conjugated to horseradish peroxidase. Nuclei were stained with DAPI after all the human antigens were labeled. The following antibodies were used. ADH1B (Proteintech, Cat# 19899-1), DPT (Proteintech, Cat# 10537-1), DCN (Signalway Antibody, Cat# 32376), POSTN (Proteintech, Cat# 19899-1), COL1A1 (Sevicebio, Cat# GB114197), ACTA2 (Sevicebio, Cat# GB13044), Keratin 14 (Sevicebio, Cat# GB11803), TEM1/CD248 (Abcam, Cat# ab204914), CD34 (Sevicebio, Cat# GB113798), PLAUR(Abcam, Cat# ab221680), IL1R (Signalway Antibody, Cat# 41670), CLDN1 (Sevicebio, Cat# GB12032), MKI67 (Sevicebio, Cat# GB111499). Masson’s trichrome staining was performed by Servicebio Technology Co., Ltd. The sections were visualized using the Pannoramic MIDI (3DHISTECH) according to the manufacturer’s protocol. Quantification of fluorescent images was performed using consistent image settings in ImageJ [84].

### Statistical analysis

Statistical analysis was carried out using Graphpad Prism 9.5 software. Wilcoxon signed-rank test was used for comparison of the differences of cell percentages between paired naïve and treatment samples, and Mann–Whitney U test for comparison of the differences of unpaired samples. A *P* value < 0.05 from a two-tailed test was < 0.05 was considered significant. * *P* < 0.05, ** *P* < 0.01, *** *P* < 0.001, **** *P* < 0.0001 or ns (not significant).

## Electronic supplementary material

Below is the link to the electronic supplementary material.

Supplementary Material 1

Supplementary Material 2

Supplementary Material 3

Supplementary Material 4

Supplementary Material 5

Supplementary Material 6

Supplementary Material 7

Supplementary Material 8

## Data Availability

All single-cell sequencing raw data of this study are deposited and available in Genome Sequence Archive (GSA, https://ngdc.cncb.ac.cn/gsa-human) under the accession number PRJCA016745. The scRNA dataset matrix accession number: OMIX005710. All software and code used in this paper are freely or commercially available. The data and other resources used in this study are available from Q.L. (the corresponding author) upon reasonable request.

## Abbreviations

apCAFs: Antigen-presenting CAFs
CAF: Cancer-Associated Fibroblast
CD248: Endosialin
CFD: Complement factor D
DC: Dendritic cell
EC: Esophageal cancer
ECM: Extracellular matrix
ESCC: Esophageal squamous-cell carcinoma
IL-6: Interleukin 6
iCAFs: Inflammatory CAFs
IM-CAF: Immunomodulatory (IM) CAF
ILC: Innate lymphoid cell
myCAFs: Myofibroblastic CAFs
MR: Mechanoresponsive
MP: Mononuclear phagocytes
NECTIN2: Nectin Cell Adhesion Molecule 2
NeoICT: Neoadjuvant immunotherapy combined with chemotherapy
PCA: Principal component analysis
RCC: Renal cell carcinoma
scRNA-seq: Single cell RNA sequencing
SSL: Steady-state-like
TME: Tumor microenvironment
TF: Transcription factor
