# Supplementary material for: Functional heterogeneity of cancer-associated fibroblasts with distinct neoadjuvant immunotherapy plus chemotherapy response in esophageal squamous cell carcinoma
Source: Biomark Res. 2024 Sep 27;12:113. doi: 10.1186/s40364-024-00656-z (PMC11437904; doi:10.1186/s40364-024-00656-z)
Supplement: Supplementary file 8 — Supplementary Material 8 [file 40364_2024_656_MOESM8_ESM.docx]

**Functional** **Heterogeneity of Cancer-Associated Fibroblasts with Distinct Neoadjuvant Immunotherapy plus Chemotherapy Response in Esophageal Squamous Cell Carcinoma**

Jun Jiang^#,1,2^, Chao Xu^#,2^, Donghui Han^#,2^, Yuan Lu^3^, Fa Yang^2^, Jiawei Wang^4^, Xiaolong Yan^5^, Xiaorong Mu^6^, Jipeng Zhang^5^, Chenghui Jia^7^, Xinyao Xu^8^, Kui Liu^1^, Zhenhua Liu^1^, Li Gong*^,6^, Yi Wan*^,1^, Qiang Lu*^,5^

^1^Department of Health Service, Base of Health Service, Air Force Medical University, Xi’an, China.

^2^Department of Urology, Xijing Hospital, Air Force Medical University, Xi'an, China.

^3^Department of Respiratory and Critical Care Medicine, Zhongda Hospital, Southeast University, Nanjing, China

^4^Department of Clinical Immunology, PLA Specialized Research Institute of Rheumatology & Immunology, Xijing Hospital, and National Translational Science Center for Molecular Medicine, Air Force Medical University, Xi'an, China

^5^Department of Thoracic Surgery, Tangdu Hospital, Air Force Medical University, Xi’an, China.

^6^Department of Pathology, Department of Pharmacy, Tangdu Hospital, Air Force Medical University, Xi’an, China

^7^Department of Thoracic Surgery, The First Affiliated Hospital of Xi'an Medical College, Xian, China

^8^College of Life Sciences, Northwest University, Xian, China

^#^ These authors have contributed equally to this work

**Corresponding author**

Qiang Lu, Department of Thoracic Surgery, Tangdu Hospital, Air Force Medical University, NO. 569 Xinsi Road, Xi’an 710038, China. Email: [luqiang@fmmu.edu.cn](mailto:luqiang@fmmu.edu.cn).

Yi Wan, Department of Health Services, Fourth Military Medical University, No.169 West Changle Road, Xi’an, 710032, China. Email: wanyi@fmmu.edu.cn

Li Gong, Department of Pathology, Department of Pharmacy, Tangdu Hospital, Air Force Medical University, NO. 569 Xinsi Road, Xi’an 710038, China. Email: lotus1909@126.com

**Contents**

**Supplemental Figure S1 scRNA-seq captures a great diversity of cell types.**

**Supplemental Figure S2 UMAP of six major stromal types and their subtypes derived from patients with ESCC**

**Supplemental Figure S3 Macrophage heterogeneity in ESCC.**

**Supplemental Figure S4 Characterization of EC in pre- and post-neoICT ESCC**.

**Supplemental Figure S5 CAF heterogeneity in ESCC**.

**Supplemental Figure S6 T cell heterogeneity in ESCC**.

**Supplemental Figure S7 Co-inhibitory checkpoint pairs in pCR and non-pCR.**


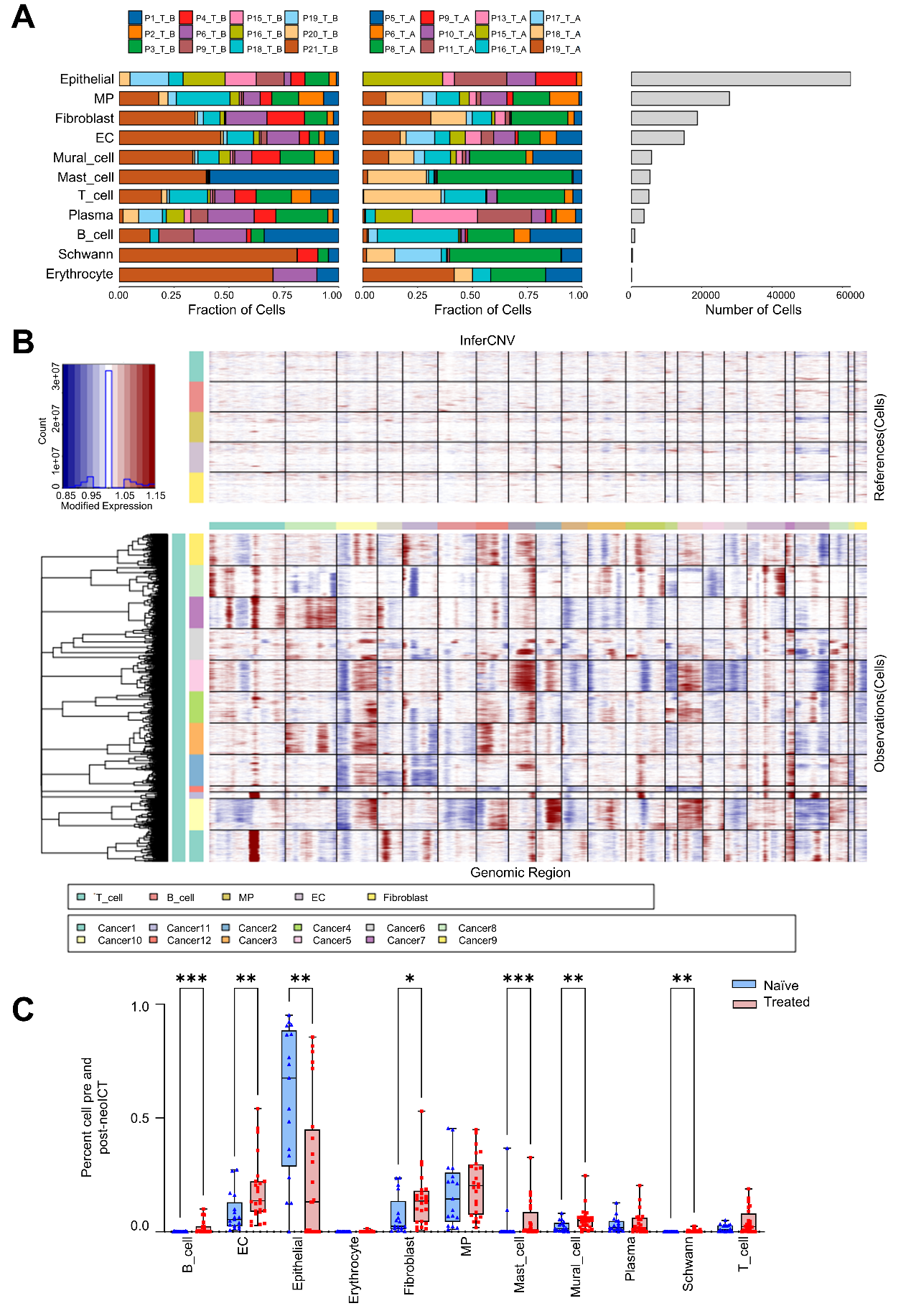


**Supplemental Figure S1 scRNA-seq captures a great diversity of cell types.** (A) The frequency of each cell type in each of the naïve and treated tumor specimens. (B) CNV profile in cancer versus stromal cells assessed using InferCNV based on scRNA-seq. CNV copy number variation，EC endothelial cell, MP, mononuclear phagocyte. (C) Comparison by Mann-Whitney U test of pre- versus post-neoadjuvant combination therapy cluster proportions. * *P* < 0.05, ** *P* < 0.01, *** *P* < 0.001. Source data are provided as a Source Data file.


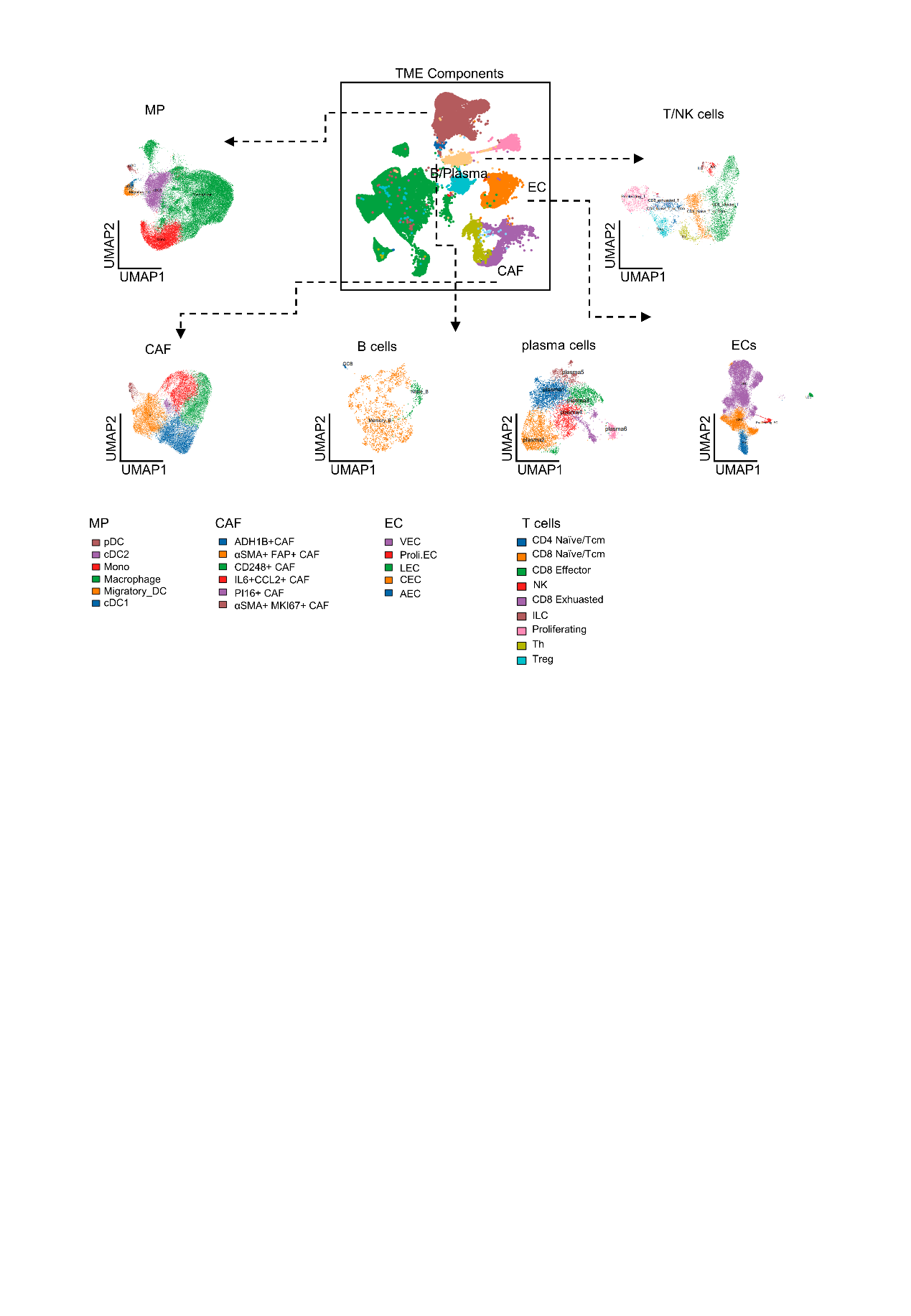
**Supplemental Figure S2 UMAP of six major stromal types and their subtypes derived from patients with ESCC**


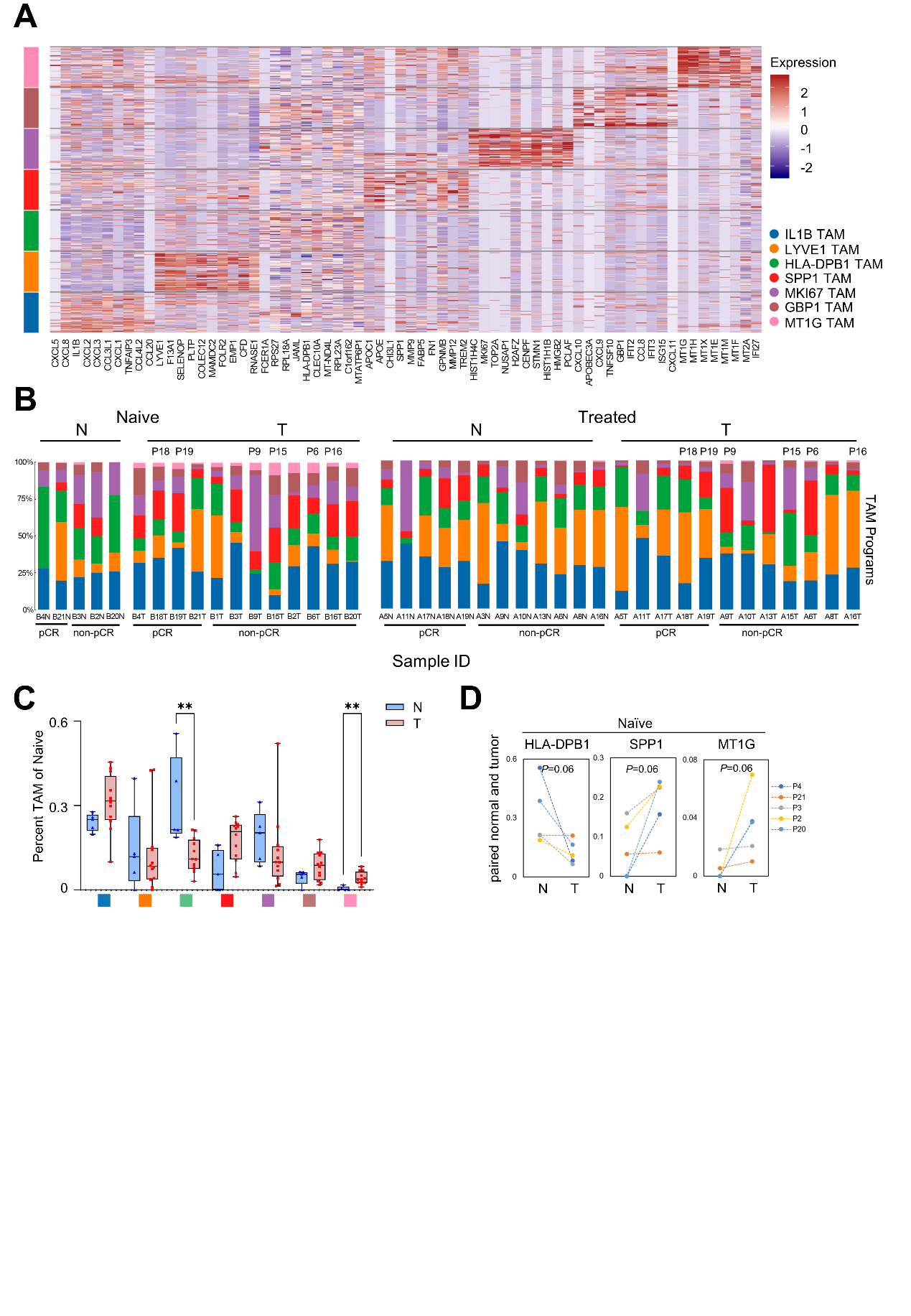
 **Supplemental Figure S3 Macrophage heterogeneity in ESCC.** (A) Heatmap showing the expression of top 10 most variable genes across each macrophage subset. (B) Macrophage cell-type distributions stratified by treatment, tissue type and pathological response across 41 samples. Proportions (y axis) of cell subsets (color legend, shared with panel a) across naive (n=17) (left) versus treated (n=24) (right). (C) Comparison of macrophage cluster proportions in naïve samples, p values determined by Mann-Whitney U test, ** *P* < 0.01. Color legend, shared with panel A. (D) Comparison of HLA-DBP1, SPP1 and MT1G subsets percent in 6 paired naïve and treated patients by Wilcoxon signed-rank test. Source data are provided as a Source Data file.


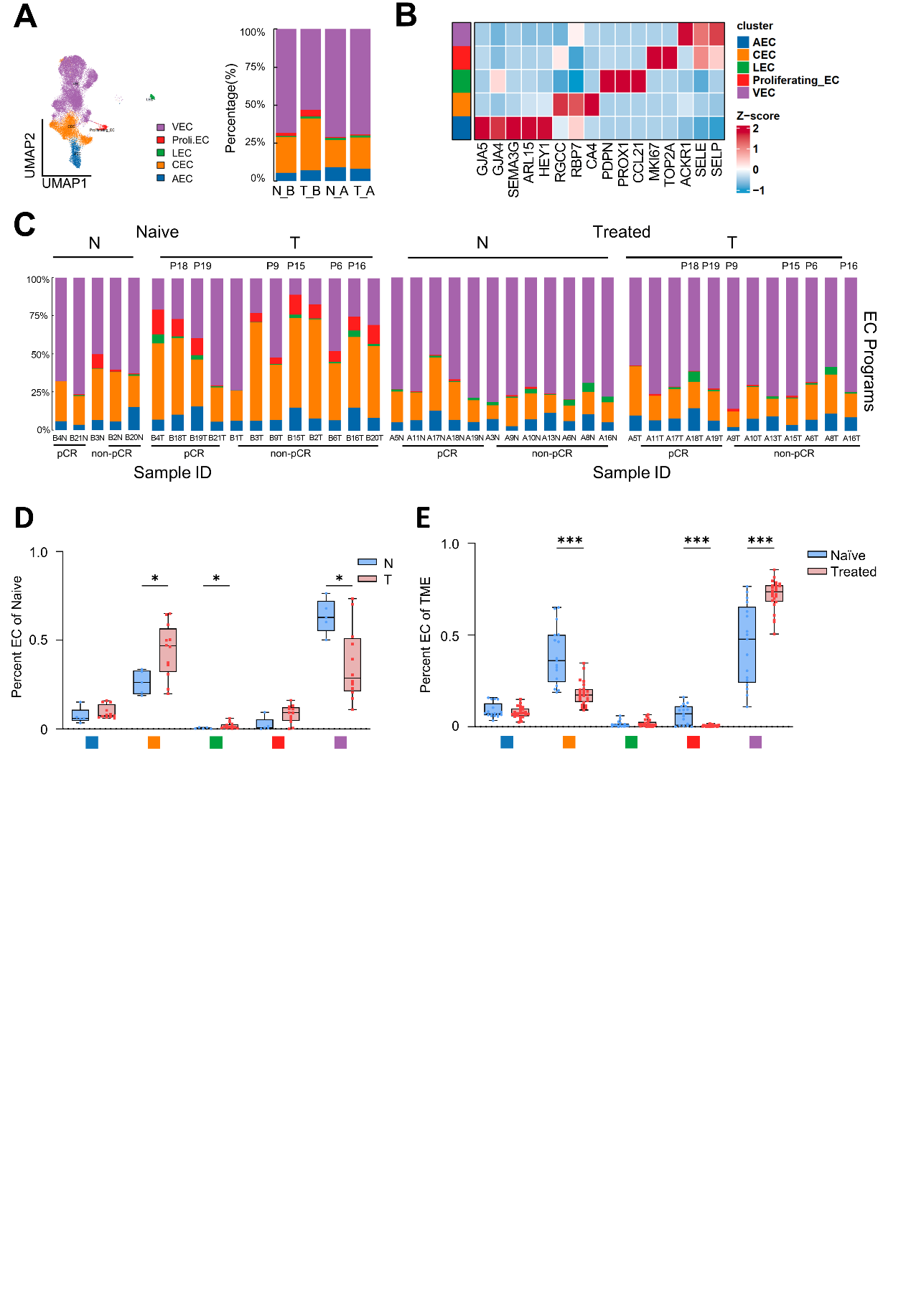
 **Supplemental Figure S4 Characterization of EC in pre- and post-neoICT ESCC**. (A) UMAP plot of sub-clusters of endothelial cells (left). The proportion of each cluster split by treatment status and tissue type (right). EC endothelial cell, VEC venous EC, LEC lymphatic EC, AEC, arterial EC. (B) Heatmap showing the expression of top 5 most variable genes across each EC subset. (C) EC subset distributions stratified by treatment, tissue type and pathological response across 41 samples. Proportions (y axis) of cell subsets across naive (n=17) (left) versus treated (n=24) (right). Color legend as in (A). (D) Comparison of EC subsets of adjacent normal (N) vs Tumor (T) in naïve samples by Mann-Whitney U test, * *P* < 0.05. Color legend as in (A). (E) Comparison of EC subsets percent in naïve and treated tumor samples by Mann-Whitney U test, *** *P* < 0.001, **** *P* < 0.0001. Color legend as in (A). Statistical plots of cell ratio are provided as a Source Data file.


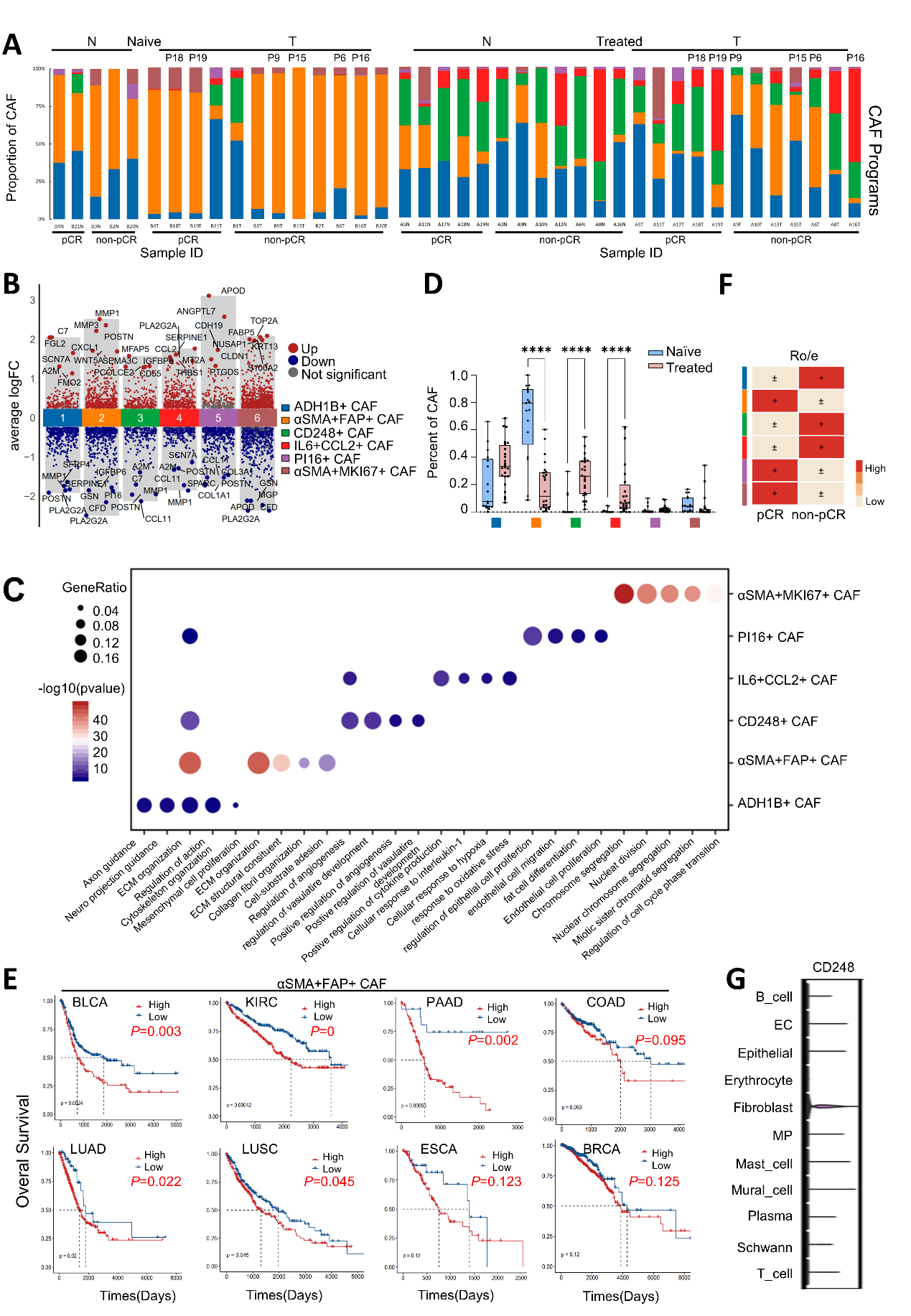


**Supplemental Figure S5 CAF heterogeneity in ESCC**. (A) CAF subsets distribution across 41 samples. Proportions (y axis) of cell subsets across untreated (n=17) (left) versus treated (n=24) tumor or adjacent normal tissues (right). Color as in (B). (B) Volcano plot showing the top five DEGs that were either up- or down-regulated across each CAF cluster. (C) Dotplot showing the indicated GO signatures in six CAF clusters. GO gene ontology. (D) Comparison of CAF subsets of adjacent normal (N) vs Tumor (T) in naïve samples by Mann-Whitney U test, * *P* < 0.05. Color as in (B). (E) Kaplan-Meier survival curves in The Cancer Genome Atlas (TCGA) dataset. BLCA Bladder Urothelial Carcinoma, KIRC Kidney Renal Clear Cell Carcinoma, PAAD Pancreatic Adenocarcinoma, COAD Colon adenocarcinoma, LUAD Lung Adenocarcinoma, LUSC Lung Squamous Cell Carcinoma, ESCA Esophageal Carcinoma, BRCA Breast Invasive Carcinoma. (F) The STARTRAC-dist index of CAF sub-cluster in treated split by pathological response. (G) Violin plot depicts CD248 transcripts in 11 cell clusters of all samples.

**
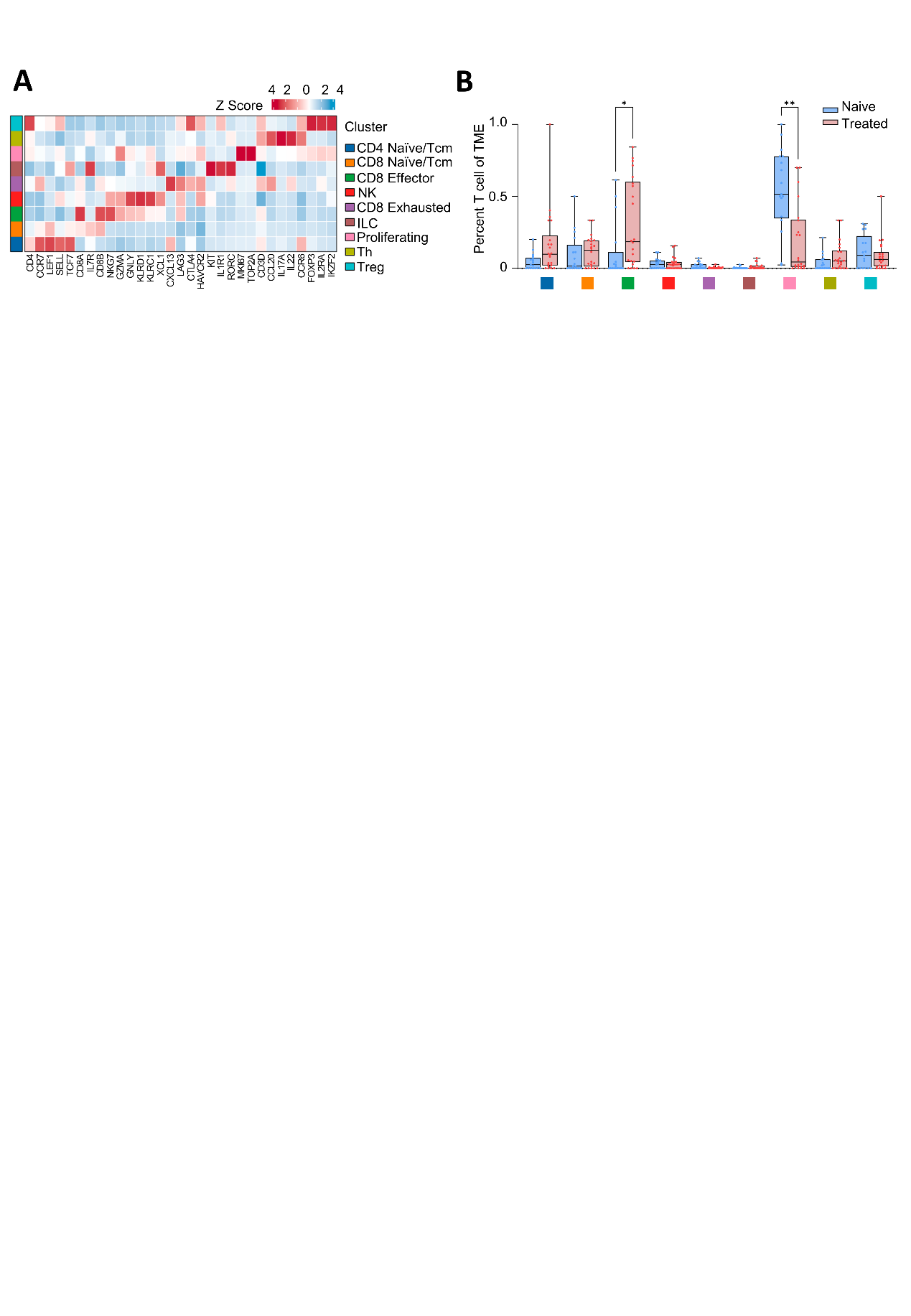
Supplemental Figure S6 T cell heterogeneity in ESCC**. (A) Heatmap showing the expression of top 5 most variable genes across each T cell subset. (B) Comparison of T subsets in tumor samples pre- and post-neoICT. *P* values determined by Mann-Whitney U test, * *P* < 0.05. Source data are provided as a Source Data file.
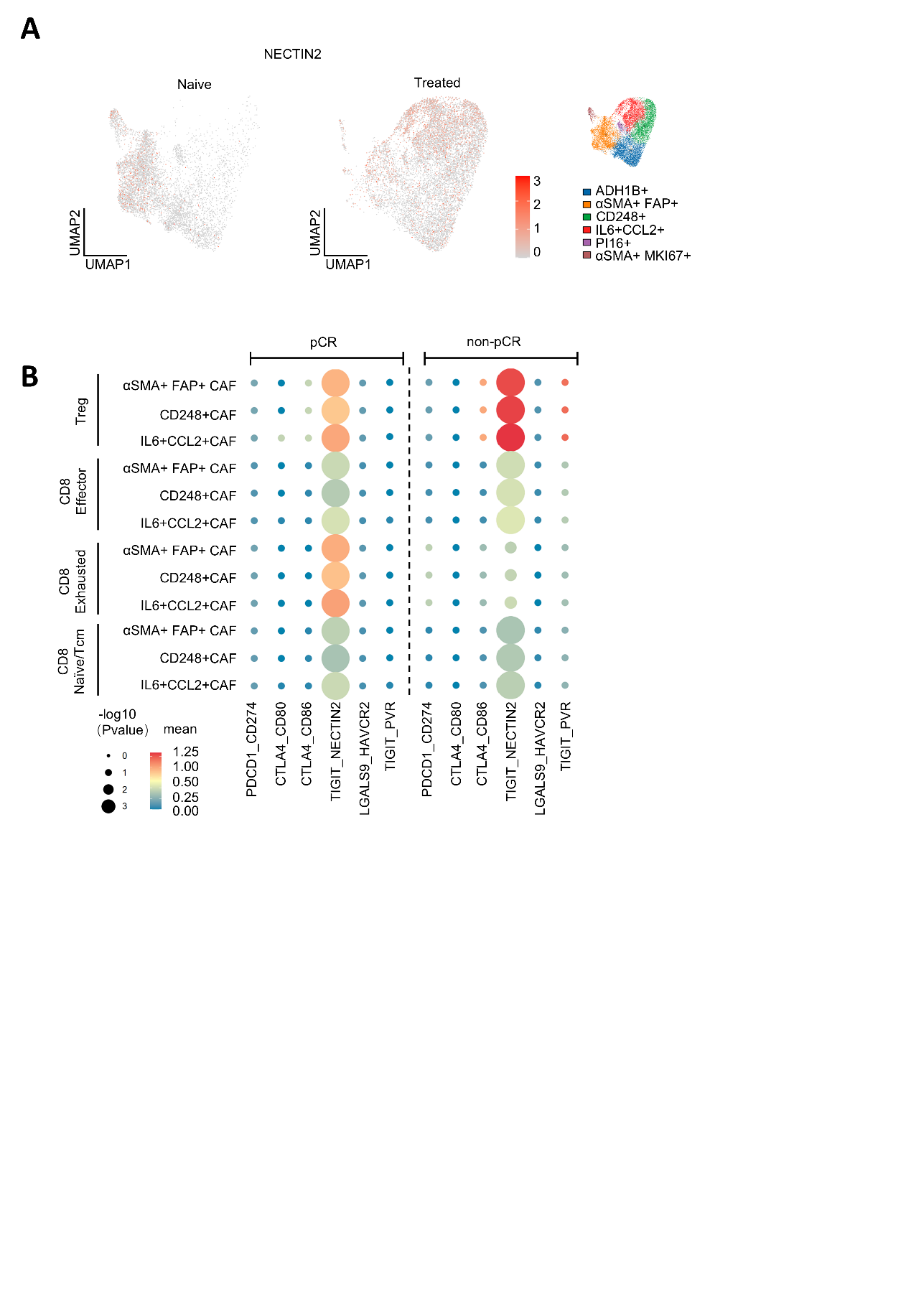
**Supplemental Figure S7 Co-inhibitory checkpoint pairs in pCR and non-pCR.** (A)UMAP plot of expression levels of NECTIN2 in CAF in treatment-naïve and treated ESCC patients. (B) Dotplots of CellphoneDB output (see Methods) showing significance (-log10 P value) and strength (mean value) of checkpoint molecule ligand-receptor interactions between CAF and T cells comparing pCR and not-pCR samples. Source data are provided as a Source Data file.
